# Trends and correlates of smokeless tobacco use among Indian women: Evidence from three rounds of the National Family Health Survey 2005–2021

**DOI:** 10.1371/journal.pone.0353704

**Published:** 2026-08-03

**Authors:** Manish Barik, Sushree Nibedita Panda, Jayanta Kumar Bora, Arpit Gupta, Sonu Goel

**Affiliations:** 1 Independent Researcher, New Delhi, India; 2 The George Institute for Global Health, Hyderabad, India; 3 VART Consulting Pvt. Ltd, Delhi-NCR & Mumbai, India; 4 Institute for Sociology and Demography, University of Rostock, Rostock, Germany; 5 German Center for Neurodegenerative Diseases (DZNE), Demographic Studies, Bonn, Germany; 6 Associate Professor, Oral Health Sciences Centre, Post Graduate Institute of Medical Education and Research (PGIMER), Chandigarh, Punjab, India; 7 Professor of Health Management, Department of Community Medicine and School of Public Health, Post Graduate Institute of Medical Education and Research (PGIMER), Chandigarh, India; 8 Adjunct Clinical Associate Professor, Public Health Master’s Program, School of Medicine, University of Limerick, Ireland; All India Institute of Medical Sciences, INDIA

## Abstract

**Background:**

Tobacco use is one of the major threats to public health on a global scale. Smokeless Tobacco (SLT) poses a significant risk for oral cancer and has other adverse health outcomes in women. This study aimed to compare the trends of smokeless tobacco use among women of reproductive age in India as well as to identify the factors associated with SLT use.

**Methodology:**

Only women aged 15–49 years of three rounds of the National Family Health Survey (NFHS), NFHS 3 (2005–2006) (n  = 1,24,385), NFHS 4 (2015–2016) (n  = 6,99,686) and NFHS 5 (2019–2021) (n = 7,24,115) were analysed separately. Correlates of SLT exposure were assessed separately using Multivariable logistic regression analysis.

**Results:**

The prevalence of SLT use among women in India declined from 8.4% in NFHS 3 to 5.6% in NFHS 4 and 3.8% in NFHS 5. Gutkha/ Paan masala was the most commonly used SLT among women in NFHS 4 (2.2%) and NFHS 5 (1.3%). In the recent NFHS 5, Mizoram had the highest (45%) prevalence of SLT use. Across the surveys lower education, older age, caste and region were found to be associated with the SLT use. Interestingly, the exposure to smoking in the house evolved as a strong predictor of SLT use in NFHS 4 [AOR 1.82(1.68-1.98)] and NFHS 5 [AOR 1.85(1.70-2.02)].

**Conclusion:**

Educational status, age, caste and exposure to household smoking were associated with SLT use. Geographical variations for SLT usage were noted among women. There is an urgent need to strengthen efforts in the high prevalent regions to prevent the initiation of smokeless tobacco (SLT) use and to support its cessation. Since India hosts the majority of the world’s SLT users, these measures could significantly impact global morbidity and mortality associated with SLT use.

## Introduction

“Smokeless tobacco" (SLT) refers to materials that can be chewed, spitted, dipped, snuffled, or applied to the teeth and gums without igniting [1]. More than 248 million people use smokeless tobacco (SLT) worldwide, 90% of users are from the Indian subcontinent [2]. SLT usage among adolescents and adults is a global concern and poses challenges in more than 100 nations. The majority of tobacco users usually start smoking during adolescence, and adolescents are more vulnerable to the harmful effects of tobacco products use because they are prone to addiction to the nicotine in tobacco [3]. While smoking tobacco is more common among men, available evidence shows that young girls and women prefer SLT over tobacco use [4]. The widespread use of SLT among women is attributed by behavioural science to socio-cultural norms and SLT’s social acceptability. Evidence highlight that SLT consumption in India is deeply embedded in traditional practices, with use often normalized within certain communities and social groups [5]. Gender norms also play a role, with SLT use being more socially acceptable among women in rural areas compared to smoking [6,7]. In India, 12.8% of women(aged ≥15 years) habitually use smokeless tobacco either daily or occasionally, reported by the Global Adult Tobacco Survey 2016–17 (GATS–2) from the Ministry of Health and Family Welfare [8]. Smokeless tobacco receives less policy attention than smoked tobacco despite its large health burden [9,10]. Evidence shows that many tobacco control policies historically focused on smoking, while SLT regulation and programmatic responses remain comparatively limited [10]. Gender responsive approaches in tobacco control remain limited, even though women constitute a substantial proportion of SLT users and require targeted prevention and cessation strategies [9,10].

SLT products are largely unregulated and under-reported [11]. Information on the composition, manufacturing, ingredients, and health risks of these preparations is scarce in India [8]. Regular SLT use has been linked to several harmful health effects, including heart disease, osteoporosis, oropharyngeal cancers, and reproductive morbidities like infertility and pregnancy complications [12–14]. Previous studies from India have shown that smokeless tobacco use among women is influenced by multiple socio-demographic and behavioural determinants [15,16]. Despite this evidence, nationally representative trend analyses focusing specifically on determinants of SLT use among Indian women across multiple NFHS rounds remain limited.

It is essential to comprehend the burden and patterns of SLT use among women to formulate gender-based tobacco control policies and programs. The changing trends and burden of SLT usage must be tracked over time.

Therefore, the present study was planned to compare the trends of smokeless tobacco use among women of reproductive age (15–49) in India using three rounds of a nationally representative sample data in the National Family Health Survey (NFHS) and to determine the factors associated with SLT use.

## Methodology

We conducted the analysis using three rounds of the “National Family Health Survey” [17] NFHS 3 (2005–2006), NFHS 4 (2015–2016), and NFHS 5 (2019–2021). These surveys provide essential data on various aspects at national, state, and district levels, including demographics, socioeconomic characteristics, family planning, maternal and child health. The Ministry of Health and Family Welfare (MoHFW) led the surveys, managed by the International Institute of Population Sciences (IIPS), Mumbai. The surveys employed a multi-stage stratified sampling design and computer-assisted personal interviews (CAPI) for data collection, ensuring geographic representation in the sampled population based on probability sampling theory.[17]

### Study participants and sample size

In this study we have analyzed data on women of reproductive age (15–49 years) derived from NFHS 3–5 to assess national-level estimates of various types of tobacco use and socio-economic and demographic correlates of smokeless tobacco use. Study sample consisted of 124,385 from NFHS 3, 699,686 from NFHS 4 and 724,115 from NFHS 5.

### Outcome variable

Participants were asked about the type of tobacco products used, with response options including khaini, gutkha, paan masala with tobacco, and paan with tobacco. Women who reported using any of these smokeless tobacco products were categorized as ‘Smokeless Tobacco (SLT) users, while those reporting no use of these products were categorized as non-users.

### Independent variables

The independent variables to include were identified through previous literature on predictors and determinants of SLT use in India [18–23]. The included independent variables were socio-demographic factors (age, religion, caste, place of residence, region, education, occupation, household wealth status, and marital status); nutritional and behavioural factors (body mass index (BMI), cigarette/tobacco smoking, alcohol consumption and household smoking status).

In this study, the self-reported age of the women was grouped into the following age groups: “15–24 years”, “25–34 years”, and “35–49 years”. Religion was classified as “Hindu”, “Muslim”, “Christian” while Sikh, Buddhist/Neo-Buddhist, Jain, Jewish, Parsi/Zoroastrian, and no religion were clubbed into “others”. Caste was labelled as “Scheduled Caste (SC)”, “Scheduled Tribe (ST)” and “Other Backward Class (OBC)”, but “None of them” and “don’t know” were merged into “Others”.

The residence of the study participants was defined as “Rural” and “Urban”. Region of India was classified as “North”, “Central”, “East”, “North-East”, “West”, and “South” based on their geographical location. Educational attainment was categorized as “No formal education” (those who never attended school), “Primary” (up to 5^th^ grade), “Secondary” (up to grade 10), and “Higher” (above secondary level). The occupation was categorized as “Professional/Sales/Services” (included technical, managerial, sales, and clerical), “Manual” (included skilled and unskilled), “Agriculture”, “Others” (other, don’t know) and “Not working”. In this study, Household wealth status was grouped as “Poorest” and “Poorer” “Middle”, and “Richer” and “Richest”. Participant’s marital status was reclassified as “Married”, “Never married” (never in union) and “Separated” (widowed, divorced, no longer living together/separated”). BMI was calculated as the ratio of weight (in kilograms) to the square of height (in meters); it was recorded as a continuous variable and was available in the three rounds of surveys. BMI was categorized as “Underweight” (less than 18.5 kg/m^2^), “Normal” (18.5–24.99 kg/m^2^), “Overweight” (25.0–29.99 kg/m^2^), and “Obese” (≥30.0 kg/m^2^) [24]. Smoking status was categorized as a female who smoked any of these (cigarettes/pipe/cigars/bidis/hookah) is labelled as “Yes” and who doesn’t smoke “No”. Exposure to passive smoking in the household in the last 30 days is categorized as “Yes” and “No”. Although efforts were made to maintain comparability across NFHS rounds, some variables were not uniformly available in all three surveys. For example, household passive smoking exposure data were available only in NFHS-4 and NFHS-5 and therefore were included only in analyses for those survey rounds. Accordingly, comparisons across survey years should be interpreted with caution for variables that differed in availability or measurement.

### Statistical analysis

Data were analyzed using STATA version 16.0 (STATA Corp., Texas) and “Microsoft Office” was used to generate figure. Descriptive statistics were used to report the frequency and proportions of socio-demographic, nutritional and health behavioural characteristics of the participants and Smokeless tobacco use. Participants with missing data on smokeless tobacco use or covariates included in the regression analyses were excluded. Multivariable logistic regression model was conducted to estimate adjusted odds ratios (AORs) with 95% confidence intervals (CI). For both descriptive and regression models, sampling weights were considered during analysis.

## Results

### Characteristics of the study population

The present study was carried out on the last three rounds of NFHS, i.e., NFHS 3 (2005−2006), NFHS 4 (2015−2016), and NFHS 5 (2019−2021). The mean age of female participants in NFHS-3 was 29.15 (SD ± 9.4) years, 29.83(SD ± 9.7) years in NFHS-4, and 30.39 (SD ± 9.8) years in NFHS-5. More than two thirds of the population were from the rural area. Nearly 90% of the participants were following Hinduism S1 Table in S1 File.

### Prevalence and types of SLT use among women

**Fig 1:**
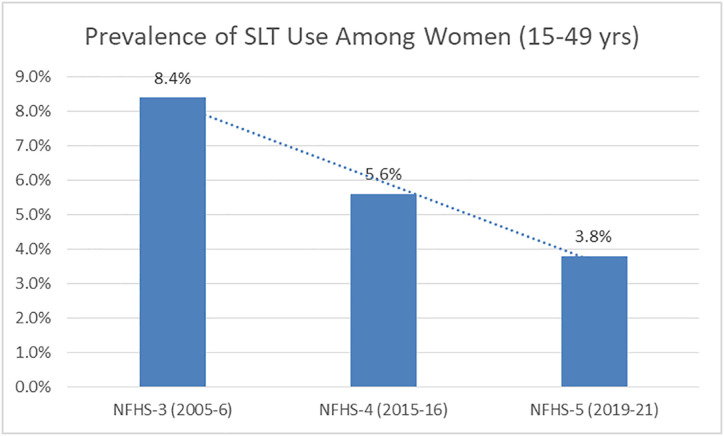
Trend of SLT use among the female population from 2005−06 to 2019−21 in India.

We observed in **Fig 1**, the gradual decline in the prevalence of smokeless tobacco uses among women across the three NFHS survey rounds, decreasing from 8.4% in NFHS-3 to 5.6% in NFHS-4 and 3.8% in NFHS-5. Although the absolute percentage reduction was modest, the findings indicate a consistent downward trend over time. During NFHS 3, chewing tobacco was the most commonly used product at 5.5%, while Gutkha or Paan masala at 1.8 percent and Paan with tobacco at 1.8% showed equal prevalence. During NFHS-4, Gutkha/ Paan masala (2.2%) was more prevalent and snuff (0.1%) was least common. Gutkha/ Paan masala (1.3%) was the most often used smokeless tobacco product in NFHS-5 whereas chewing tobacco (0.3%) had minimal use. S1 Table in S1 File has the detailed frequency of several types of SLT products used.

### Distribution of SLT use among women

Supplementary table 1 in S1 File shows that across all three NFHS rounds, the majority of female smokeless tobacco users belonged to the 35–49 years age group. In both rural and urban settings, the prevalence of SLT use among women declined in NFHS-5 compared with NFHS-3. However, compared with NFHS-4, a relatively higher prevalence of SLT use was observed among urban women in NFHS-5. The majority of female SLT users had primary or no education, while the number of female SLT users with primary education was noticeably greater in NFHS-5 than in NFHS-3 and 4. Participants with agricultural occupations had a higher prevalence of SLT use in NFHS 5, despite a downward trend in SLT usage. According to this study, SLT use was more widespread among women from the lowest socioeconomic backgrounds (8.3%) in NFHS-5 than in the previous two rounds of NFHS. This study explained that the prevalence of SLT usage was higher among women who were separated (8.7%) in NFHS-5 although a downward trend was found in the last three rounds of NFHS. Though the overall prevalence has decreased, a spike in the North-eastern female population has been found, with SLT use increasing from 5.6% in NFHS-4 to 17.6% in NFHS-5 which was previously lower in NFHS-4 than NFHS-3(27%). In NFHS-5, the Scheduled Tribe group had a greater percentage of SLT use (10.3%). According to current SLT prevalence by religion, Christian women (6.8%) were user of SLT in NFHS-5 despite the fact that SLT use is declining across the board in the previous two rounds of NFHS. The prevalence of SLT usage was higher among underweight women (4.1%) in NFHS-5, even though SLT use was dropping in all subgroups in the previous two rounds of NFHS. SLT was used by 5.1% of women participants in NFHS-5 who were exposed to passive smoking in their households which was lower than NFHS-4. Interestingly, in all the last three rounds of NFHS, there is a decrease in SLT usage among women who smoke.

### State-wise prevalence of SLT use in NFHS 5

Substantial geographical variation in SLT use was observed across Indian states, with particularly higher prevalence reported in several North-Eastern states. Mizoram had the highest prevalence of 45%, followed by Manipur at 41.4%, while Chandigarh followed by Punjab had less than 1% and Himachal Pradesh at 0.09% (S1 Fig in S1 File).

### Factors associated with SLT use

Multivariable regression analysis Table 1 revealed that the likelihood of SLT usage was [Adjusted Odds Ratio (AOR):1.91(1.47-2.09)] in 25–34 years and [AOR:3.22(2.93-3.52)] in 35–49 years age categories during the NFHS 3 survey to [AOR: 1.71 (1.51-1.94)] in 25–34 years and [AOR: 2.82 (2.50-3.19)] in 35–49 years during NFHS 4, subsequently higher during NFHS 5 survey [AOR:1.87 (1.58-2.21) in 25–34 years and [AOR:2.93 (2.48-3.47)] in 35–49 years. Those who were from the Muslim community [AOR 1.90(1.43-2.53)] showed a higher likelihood of the SLT use compared to “others” religious category in NFHS 5. Although there was no change in the caste association, STs had a higher likelihood of using SLT in all three rounds of the survey than the other social categories. In the last three rounds, the North-Eastern women had the highest chance of using SLT [AOR: 12.28(10.94-13.80)] during NFHS-3, [AOR 15.17 (13.03-17.65)] during NFHS 4 and [AOR 15.99(13.28-19.26)] during NFHS 5. Women from Northern and Western parts of India had association with SLT usage in NFHS 5 than NFHS 3, but the Eastern States of India the likelihood of SLT usage was [AOR 3.52(3.15-3.92)] in NFHS 3 vs. [AOR 2.93(2.43-3.54)] in NFHS 5.

**Table 1:** Multivariable logistic regression analysis depicting the association of SLT use among women (15-49 years) with various socio-demographic, behavioural and nutritional characteristics in NFHS 3, NFHS 4 and NFHS 5.

	NFHS 3	NFHS 4	NFHS 5
Socio-demographic characteristics	Adjusted OR (95% CI)	Adjusted OR (95% CI)	Adjusted OR (95% CI)
**Age group (in years)**			
15-24	Ref	Ref	Ref
25-34	1.91***(1.47-2.09)	1.71***(1.51-1.94)	1.87*** (1.58-2 21)
35-49	3.22***(2.93-3.52)	2.82*** (2.50-3.19)	2.93*** (2.48-3.47)
**Religion**			
Hindu	1.03(0.85-1.23)	1.45** (1.14-1.85)	1.52**(1.19-1.95)
Muslim	1.22*(1.00-1.50)	1.76***(1.35-2.30)	1.90*** (1.43-2.53)
Christian	0.80(0.64-1.00)	1.19 (0.91-1.56)	1.39*(1.1-1.8)
Others	Ref	Ref	Ref
**Caste**			
Scheduled Caste	1.17**(1.07-1.28)	1.30***(1.13-1.50)	1.18*(1.01-1.39)
Scheduled Tribe	2.13***(1.92-2.35)	1.93***(1.67-2.23)	2.12*** (1.80-2.48)
Other Backward Class	0.72***(0.66-0.79)	0.92(0.80-1.04)	0.83*(0.72-0.96)
Others	Ref	Ref	Ref
**Residence**			
Rural	0.74*** (0.68-0.80)	0.66, *** (0.59-0.74)	0.73*** (0.64-0.83)
Urban	Ref	Ref	Ref
**Region**			
North	0.64***(0.54-0.76)	0.99(0.83-1.18)	1.68*** (1.38-2.06)
Central	3.30***(2.96-3.67)	2.50***(2.17-2.87)	3.21***(2.70-3.81)
East	3.52***(3.15-3.92)	2.25***(1.93-2.62)	2.93***(2.43-3.54)
North-east	12.28***(10.94-13.80)	15.17***(13.03-17.65)	15.99***(13.28-19.26)
West	2.19***(1.93-2.49)	3.12***(2.63-3.69)	4.18***(3.43-5.09)
South	Ref	Ref	Ref
**Education**			
No formal education	5.19***(4.03-6.69)	9.05***(6.93-11.83)	7.56***(5.43-10.52)
Primary	5.36***(4.16-6.92)	8.00***(6.18-10.36)	7.98***(5.73-11.11)
Secondary	2.97***(2.33-3.79)	3.95***(3.12-4.99)	3.66***(2.66-5.05)
Higher	Ref	Ref	Ref
**Occupation**			
Professional/Sales/Services	1.65***(1.48-1.85)	1.65***(1.41-1.92)	1.50***(1.28-1.76)
Manual worker	1.72***(1.57-1.88)	1.64***(1.44-1.86)	1.90***(1.63-2.21)
Agriculture	1.32***(1.22-1.42)	1.40***(1.28-1.54)	1.67***(1.50-1.86)
Not working	Ref	Ref	Ref
**Marital status**			
Never married	Ref	Ref	Ref
Married	1.83***(1.62-2.07)	1.84***(1.57-2.17)	1.90***(1.57-2.31)
Separated	2.25(1.91-2.65)	2.71***(2.20-3.36)	3.13***(2.45-3.99)
**Body Mass Index (Kg/M**^**2**^)			
Normal (18.5-24.9)	1.13(0.89-1.43)	1.12, (0.90-1.40)	1.13(0.88-1.44)
Underweight (<18.5)	1.41**(1.10-1.79)	1.46**(1.16-1.83)	1.49**(1.16-1.91)
Overweight (25-29.9)	1.09(0.89-1.43)	0.99(0.78-1.25)	0.99(0.77-1.29)
Obese (≥ 30)	Ref	Ref	Ref
**Wealth Index of Household**			
Poorest	2.81***(2.41-3.27)	3.09***(2.42-3.93)	2.98***(2.22-4.01)
Poorer	2.18***(1.88-2.53)	2.67***(2.11-3.37)	2.32***(1.74-3.09)
Middle	2.00***(1.74-2.29)	1.95***(1.56-2.44)	2.00***(1.51-2.65)
Richer	1.61***(1.41-1.83)	1.57***(1.26-1.98)	1.29(0.97-1.72)
Richest	Ref	Ref	Ref
**Passive smoking**			
Yes	Not Available	1.82***(1.68-1.98)	1.85***(1.70-2.02)
No	Not Available	Ref	Ref
**Respondent smokes**			
Yes	1.0(0.87-1.17)	1.1(0.85-1.44)	1.06(0.66-1.73)
No	Ref	Ref	Ref


*** P < 0.001 level of significance

** P ≤ 0.01 level of significance

* P ≤ 0.05 level of significance

Ref: Reference category

Educational level emerged as an important contributing factor for SLT use. Women with no formal education had the highest odds of using SLT [AOR: 9.05 (6.93-11.83)] during NFHS 4 whereas women with primary-level education showed the highest likelihood of SLT use in NFHS 5[AOR 7.98(5.73-11.11)] as well as in NFHS 3. Compared with women who were not working, women engaged in manual work, agriculture, and professional/service occupations had significantly higher odds of smokeless tobacco use across all three NFHS rounds. Among occupational groups, manual workers consistently demonstrated the highest odds of SLT use, followed by women engaged in agricultural occupations. One notable finding of our study was that women who were separated and did not live with their partners had a higher likelihood of using SLT than women who were not married. A similar trend was found in SLT association with the economic status in all three rounds of the survey, the most deprived group showed the highest likelihood of SLT use than the affluent group [AOR 2.81(2.41-3.27) in NFHS 3, [AOR 3.09 (2.42-3.93)] in NFHS 4 and [AOR 2.98(2.22-4.01)] in NFHS 5. Compared with obese women, underweight women had significantly higher odds of smokeless tobacco use across all three NFHS rounds. Interestingly, exposure to smoking in the house evolved as a strong predictor of SLT use in NFHS 4 [AOR 1.82(1.68-1.98)] and NFHS 5 [AOR 1.85(1.70-2.02)].

## Discussion

This study assessed the nationwide prevalence and trend of SLT use and associated factors among Indian women aged 15–49 years using three rounds of the National Family Health Survey. We found the overall prevalence of SLT was consistently declining from 8.4% in NFHS 3 to 5.6% in NFHS 4 and 3.8% in NFHS 5. Although a recent systematic review across 82 low- and middle-income countries reported an overall SLT prevalence of 7.7%, direct comparison with the present findings should be interpreted cautiously due to differences in study populations, countries included, and tobacco use assessment methods [25–27]. Additionally, some previous Indian studies have reported relatively higher SLT prevalence, particularly in region-specific or high-burden populations, which may differ from the nationally representative female population included in the present study. The use of Gutkha/Paan masala was more popular among women in the last two rounds of the survey, whereas chewing tobacco was the most commonly used product in NFHS 3, which was consistent with a prior study in western India [28]. but the study carried out in India using GATS data(2017) reported Betel quid to be the most commonly used tobacco [29].

The present study indicated that the prevalence of tobacco usage increased with age; women in the 35−49 years age group had a higher prevalence in the last three rounds of the survey(2005−2021) previous literature also confirms this, may be linked to long standing social and cultural acceptance of smokeless tobacco in certain communities, particularly in rural and socioeconomically disadvantaged settings. Older women are more likely to have initiated tobacco use earlier in life when awareness of health risks and tobacco control measures were limited [8,29,30]. In NFHS 3 and 4, women from rural areas had a higher prevalence of SLT use, however, the recent round conducted in 2019−21 reported a lower prevalence among rural women while urban areas had a higher consumption rate, These changing patterns may reflect differences in tobacco control awareness, sociocultural practices, accessibility of smokeless tobacco products, and evolving consumption behaviours across rural and urban populations [31,32]. Women with no formal education and engaged in agricultural work tend to indulge more in SLT consumption. Here, there was a rise in SLT users among the lowest socioeconomic backgrounds in all the rounds of the survey, SLT is comparatively inexpensive and also the availability, accessibility and promotion of smokeless tobacco in a low-income area may also the cause of this [33]. There was an apparent surge in the prevalence of SLT use among women belonging to the North-eastern region in all the rounds of the survey. The higher prevalence of smokeless tobacco use observed in several North-Eastern states may reflect longstanding sociocultural acceptance of tobacco consumption, traditional chewing practices, easier local availability of smokeless tobacco products, and weaker enforcement of tobacco control measures reported in these regions [34]. It was found that Scheduled Tribes were the highest consumer of SLT in NFHS 5. Women belonging to the Muslim community have higher odds of using SLT, previous literature is also in concordance with the evidence [35]. This study had an interesting finding that tribal women in India are more likely to use SLT, followed by SCs.

Education is a significant predictor of SLT as this study found an association between education level and SLT consumption [36]. SLT use was more prevalent among women without a formal education. The observed differences in SLT use may reflect broader sociocultural and contextual factors that warrant further investigation. Women manual workers and women engaged in agricultural work showed higher odds of SLT usage than those who were unemployed or in any other occupation. Several studies indicated people tend to use tobacco when they need to work hard or to acquire mental relaxation [36,37]. Similar to this, the likelihood of SLT usage was higher in the poorest quintile than the richest [8,38,39]. The likelihood of using SLT is substantially increasing in Western, Northern and North-eastern part. The women residing in north-eastern region have higher odds this study results strongly affirm that SLT use is socially acceptable and integral part of the culture of the region, similar findings also reported in previous studies [40,41]. However the Eastern part of country somehow manged to reduce the burden of SLT use in last three rounds of survey. Moreover, it provides an opportunity to systematically investigate the factors for declining trend and implement relatively similar approach in other regions. One of the significant findings of this study is the women who are exposed to household smoking are more likely to use SLT than the others in last two rounds of survey and odds have increased from NFHS 4 to NFHS 5. Exposure to smoking within the household may influence women’s use of smokeless tobacco through social and behavioural mechanisms [42]. Evidence from India indicates that tobacco use among close social contacts such as family members, spouses, and friends increases the likelihood of tobacco use through social influence and normalization of the behaviour [43].

### Methodological limitations

This study examines the trends in smokeless tobacco use among women of reproductive age using recent rounds of the NFHS survey as well as the associated factors of SLT use. The self-reported nature of the data collected in NFHS which introduces the possibility of social desirability bias and underreporting of smokeless tobacco use among women due to prevailing social norms. Data collection for National Family Health Survey 5 occurred during the COVID 19 period, when heightened stigma and public health messaging against tobacco consumption influenced disclosure. As a result, the reported prevalence of smokeless tobacco use among women of reproductive age in this analysis likely represents an underestimate of the true burden. The adjusted odds ratios were estimated separately for each NFHS round and we did not fit a pooled regression model with interaction terms between survey round and key determinants to formally test changes in associations over time. Therefore, comparisons of adjusted odds ratios across rounds should be interpreted descriptively rather than as statistically tested differences. Although sampling weights were incorporated to obtain population level estimates and appropriate standard errors, we did not apply a multilevel modelling framework to explicitly account for hierarchical variation at household, cluster, or state levels. This may limit assessment of contextual effects and between cluster heterogeneity. And the cross-sectional design of NFHS restricts causal inference. Observed associations do not establish temporal or causal relationships between socio demographic factors and smokeless tobacco use. Some variables were not consistently available across all NFHS rounds, which may have limited direct comparability of certain determinants over time.

### Policy implications and recommendations

Global SLT market is anticipated to be expanded within the next few years which needs an urgent attention. In India within last few years there is a declining trend of SLT use seen among women which may be attributed by the awareness campaigns about the harms of SLT and ongoing public service announcements in cinema halls and on television. [44,45] There was the provision of new images and text warnings were to occupy 85% of pack area on both front and back from 1st April 2016, however these health warnings have generally not appeared on gutka packages, which may be related to the use of gutka remaining a major SLT product used by women. [46,47] Also the bans on certain smokeless tobacco products, such as gutka and pan masala, under the Food Safety and Standards Act 2006 and its Regulations of 2011, have not been effectively enforced in many states across India. Despite these legal restrictions, these products continue to be widely available, often sold informally in numerous shops, particularly in rural and semi-urban areas, as highlighted in the study Gutka remains a major smokeless tobacco product used by women. In order to make gender-based tobacco control strategies acceptable on a societal and cultural level, a bottom-up approach should be adopted with women’s participation. In order to raise public awareness on the tobacco lobby and the numerous tobacco control legislation, developing countries like India must engage with politicians, legislators, the media, civil society, and the general public. Community-based preventive and awareness initiatives should strive to reach both men and women equally. The “National Quit-lines” under the National Tobacco Control Programme (NTCP) should be promoted at both district and local levels, along with providing detailed information about m-Cessation. Cessation counselling services should be integrated into community health centers, and all healthcare workers should be trained to provide basic cessation services, including counselling. Nicotine replacement therapy, where needed, should be included as part of essential medicines. Furthermore, community-based research should be encouraged to better understand the barriers, challenges, and opportunities, allowing for the development of effective behavioural intervention strategies that can be integrated into community health clinics to support SLT cessation Awareness materials should be created in the local language with more illustrations to reach all social strata. Health practitioners should screen all patients visiting healthcare facilities and offer cessation advice to ensure that this opportunity is not overlooked.

## Conclusion

Over the years, tobacco control initiatives have reduced SLT use among women in India, but this development must be accelerated further to achieve the Indian health target that aims to reduce tobacco use by 15% by 2020 and by 30% by 2025.Therefore, it necessitates more focused gender-based, societal and context-specific approach to address the discrepancies in smokeless tobacco use as well as strict enforcement of tobacco control programmes and regulation. To generate concrete evidence, more specific gender-based tobacco research is need of the hour.

## Supporting information

S1 File Sample characteristics and state-wise prevalence of SLT usage. (DOCX)
