# Supplementary material for: Trends and correlates of smokeless tobacco use among Indian women: Evidence from three rounds of the National Family Health Survey 2005–2021
Source: PLoS One. 2026 Aug 3;21(8):e0353704. doi: 10.1371/journal.pone.0353704 (PMC13432099; doi:10.1371/journal.pone.0353704)
Supplement: S1 File — (DOCX) [file pone.0353704.s001.docx]

**Supplementary table 1** The sample characteristics and weighted prevalence of SLT use among women NFHS 3, NFHS 4 and NFHS 5

| **Sociodemographic characteristics** | **NFHS 3** | | **NFHS 4** | | **NFHS 5** | | **Absolute change in SLT prevalence** | |
| --- | --- | --- | --- | --- | --- | --- | --- | --- |
|  | **Total**  **n, %**  **(weighted)** | **SLT users**  **n, %,**  **(weighted)** | **Total**  **n, %**  **(weighted)** | **SLT users**  **n, %,**  **(weighted)** | **Total**  **n, %**  **(weighted)** | **SLT users**  **n, %,**  **(weighted)** | **NFHS 3 and NFHS 4** | **NFHS 4 and NFHS 5** |
| **Age group** |  |  |  |  |  |  |  |  |
| 15-24 | 47,590  (38.2%) | 1,792  (3.7%) | 2,44,517  (34.95%) | 4,975  (2.0%) | 2,41,983  (33.4%) | 2,856  (1.1%) | -1.7% | -0.9% |
| 25-34 | 38,072  (30.6%) | 3,358  (8.8%) | 2,11,811  (30.27%) | 10,984  (5.1%) | 2,17,528  (30%) | 7,435  (3.4%) | -3.7% | -1.7% |
| 35-49 | 38,721  (31.2%) | 5,280  (13.6%) | 2,43,356  (34.78%) | 23,184  (9.5%) | 2,64,602  (36.6%) | 16,838, (6.3%) | -4.1% | -3.2% |
| **Religion** |  |  |  |  |  |  |  |  |
| Hindu | 1,00,110  (80.6%), | 8,276  (8.2%) | 5,63,738  (80.57%) | 30,716 (5.4%) | 5,89,164  (81.3%) | 21,770  (3.6%) | -2.8% | -1.8% |
| Muslim | 16,928  (13.6%) | 1,539  (9.1%) | 96,461  (13.79%) | 6,063  (6.2%) | 97,595  (13.4%) | 3,664  (3.7%) | -2.9% | -2.5% |
| Christian | 3,051  (2.4%) | 318  (10.4%) | 16,619  (2.38%) | 1,506  (9.0%) | 16,995  (2.3%) | 1,164  (6.8%) | -1.4% | -2.2% |
| Others | 4,092  (3.3%) | 268  (6.5%) | 22,866  (3.27%) | 859  (3.7%) | 20,360  (2.8%) | 530  (2.6%) | -2.8% | -1.1% |
| **Caste** |  |  |  |  |  |  |  |  |
| Scheduled Caste | 22,748  (19.1%) | 2,385  (10.4%) | 1,42,341  (21.34%) | 8,977  (6.3%) | 1,58,709  (23.1%) | 6,511  (4.1%) | -4.1% | -2.2% |
| Scheduled Tribe | 9,953  (8.3%) | 2,114, (21.2%) | 64,019  (9.6%) | 8,876  (13.8%) | 67,359  (9.8%) | 6,967 (10.3%) | -7.4% | -3.5% |
| Other Backward Class | 48,085  (40.53%) | 2,827  (5.8%) | 3,03,246  (45.46%) | 12,588  (4.1%) | 3,11,226  (45.4%) | 8,102  (2.6%) | -1.7% | -1.5% |
| Others | 37,857 (31.9%) | 2,500  (6.6%) | 1,57,467  (23.61%) | 6,749  (4.2%) | 1,48,128  (21.6%) | 3,566 (2.4%) | -2.4% | -1.8% |
| **Residence** |  |  |  |  |  |  |  |  |
| Rural | 83,567  (67.2%) | 8,169  (9.7%) | 4,57,461  (65.4%) | 29,988  (6.5%) | 88,836  (67.5%) | 5,310  (2.2%) | -3.2% | -4.3% |
| Urban | 40,817  (32.8%) | 2,262  (5.5%) | 2,42,224  (34.6%) | 9,157  (3.7%) | 2,35,278  (32.5%) | 21,820  (4.5%) | -2.2% | 0.8% |
| **Region** |  |  |  |  |  |  |  |  |
| North | 16,523, (13.2%) | 332  (2%) | 95,098 (13.5%) | 2,001 (2.1%) | 1,02,199  (14.1%) | 1,765  (1.7%) | -1.5% | -1.8% |
| Central | 28,929 (23.2%) | 3,224, (11.1%) | 1,65,473 (23.6%) | 11,815 (7.1%) | 1,80,228  (24.9%) | 7,461  (4.1%) | -4.5% | -2.5% |
| East | 27,913 (22.4%) | 3431, (12.2%) | 1,54,697 (22.1%) | 10,231 (6.6%) | 1,61,828  (22.7%) | 7,038  (4.2%) | -5.1% | -2.9% |
| North-east | 4,912  (3.9%) | 1,327  (27%) | 24,615 (3.5%) | 5,961  (24.2%) | 26,745  (3.7%) | 4,713  (17.6%) | -2.8% | -6.6% |
| West | 18,442 (14.8%) | 1,222  (6.6%) | 1,00,535 (14.3%) | 5,709 (5.6%) | 1,02,036  (14.1%) | 4,152  (4%) | -1% | -1.6% |
| South | 27,663 (22.2%) | 894  (3.2%) | 1,59,266 (22.7%) | 3,425 (2.1%) | 1,48,077  (20.5%) | 1,999  (1.3%) | -1.1% | -0.8% |
| **Education** |  |  |  |  |  |  |  |  |
| No formal education | 50,485  (40.5%) | 6,809, (13.4%) | 1,92,135  (27.46%) | 21,972  (11.4%) | 1,62,459  (22.4%) | 13,572  (8.3%) | -2% | -3.1% |
| Primary | 18,287  (14.7%) | 1,814  (9.9%) | 87,232  (12.47%) | 7,329  (8.4%) | 54,922  (11.8%) | 5,817  (10.5%) | -1.5% | 2.1% |
| Secondary | 46,525  (37.4%) | 1,709  (3.6%) | 3,31,037  (47.31%) | 9,268  (2.8%) | 3,63,395  (50.2%) | 7,374  (2%) | -0.8% | -0.8% |
| Higher | 9,073  (7.3%) | 97  (1.0%) | 89,280  (12.76%) | 574  (0.6%) | 1,13,345  (15.6%) | 365  (0.3%) | -0.4% | -0.3% |
| **Occupation** |  |  |  |  |  |  |  |  |
| Professional/Sales/Services | 10,006 (8%) | 875  (8.7%) | 9,501  (7.89%) | 596  (6.2%) | 10,914  (10.1%) | 439  (4%) | -2.5% | -2.2% |
| Manual worker | 11,782  (9.5%) | 1,556  (13.2%) | 7,677  (6.37%) | 782  (10.1%) | 6,812  (6.2%) | 490  (7.1%) | -3.1% | -1.1% |
| Agriculture | 31,268  (25.1%) | 3,750  (12%) | 17,915  (14.87%) | 1,870  (10.4%) | 15,292  (14% | 1,277  (8.3%) | -1.6% | -2.1% |
| Not working | 71,226  (57.3%) | 4,237  (5.9%,) | 85,375  (70.87%) | 3,571  (4.1%) | 75,634  (69.6%) | 2,171  (2.8%) | -1.8% | -1.3% |
| **Marital status** |  |  |  |  |  |  |  |  |
| Never Married | 25,462  (20.5%) | 623  (2.4%) | 1,59,034  (22.7) | 2,444  (1.5%) | 1,72,074  (23%) | 1,422  (0.8%) | -0.9% | -0.7% |
| Married | 93,088  (74.9%) | 8.857  (9.5%) | 5,11,372  (73%) | 33,138  (6.4%) | 5,21,351, (72%) | 23,007  (4.4%) | -3.1% | -2.0% |
| Separated | 5,833  (4.6%) | 951  (16.3%) | 29,278  (4.3%) | 3,561  (12.1%) | 30,688  (4.2%) | 2,699 (8.7%) | -4.2% | -3.4% |
| **Body Mass Index (Kg/M^2^)** |  |  |  |  |  |  |  |  |
| Normal (18.5-24.9) | 46,690  (37.5%) | 4,821  (10.3%) | 3,91,205  (57.37%) | 22,035  (5.6%) | 3,99,765  (55.2%) | 15,782  (3.9%) | -4.7% | -1.7% |
| Underweight (<18.5) | 63,948  (50.6%) | 4,870  (7.7%) | 1,62,861  (23.47%) | 11,042  (6.7%) | 1,57,989  (21.8%) | 6,595  (4.1%) | -1% | -2.6% |
| Overweight (25-29.9) | 11,475  (9.2%) | 609  (5.3%) | 1,05,281  (15.17%) | 4,655  (4.4%) | 1,21,752  (16.2%) | 3,628  (2.9%) | -0.9% | -1.5% |
| Obese (≥30) | 3,271 (2.6%) | 131  (4%) | 34,692  (5%) | 1,208  (3.4%) | 44,196  (6.1%) | 1,105  (2.5%) | -0.6% | -0.9% |
| **Wealth Index of the household** |  |  |  |  |  |  |  |  |
| Poorest | 21,717  (17.4%) | 3,648  (16.8%) | 1,24,054  (17.73%) | 13,768  (11.1%) | 1,33,973  (18.5%) | 11,230  (8.3%) | -5.7% | -2.8% |
| Poorer | 23,616 (18.9%) | 2,646, (11.2%) | 1,36,900  (19.57%) | 11,336  (8.2%) | 1,44,813  (20%) | 7,384  (5.1%) | -3.0% | -3.1% |
| Middle | 25,088 (20.1%) | 1,995  (7.  9%) | 1,43,814  (20.55%) | 7,366  (5.1%) | 1,48,616  (20.5%) | 4,787  (3.2%) | -2.8% | -1.9% |
| Richer | 26,106, (20.9%) | 1,428  (5.4%) | 1,47,978  (21.15%) | 4,647  (3.1%) | 1,50,680  (20.8%) | 2,621  (1.7%) | -2.3% | -1.4% |
| Richest | 27,856, (22.4%) | 713  (2.5%) | 1,46,939  (21%) | 2,026  (1.4%) | 1,46,032  (20.1%) | 1,105, 0.07% | -1.1% | -1.3% |
| Exposed to passive smoking in the last 30days |  |  |  |  |  |  |  |  |
| Yes | # | # | 3,73,736  (53.4%) | 27,441  (7.3%) | 3,45,618  (47.73%) | 17,648 (5.1%) | # | -2.2% |
| No | # | # | 3,25,949  (46.6%) | 11,703  (3.6%) | 3,78,496  (52.2%) | 9,481  (2.5%) | # | -1.1% |
| **Respondent smokes** |  |  |  |  |  |  |  |  |
| Yes | 2,805  (2.2%) | 504  (18%) | 5,412  (0.7%) | 833  (15.4%) | 2,453  (0.3%) | 267  (10.8%) | -2.6% | -4.6% |
| No | 1,21,579  (97.7%) | 9,927  (8.1%) | 6,94,273  (99.3%) | 38,311  (5.5%) | 7,21,661  (99.6%) | 26,861, (3.7%) | -2.6% | -1.8% |
| Hypertension |  |  |  |  |  |  |  |  |
| Yes | # | # | 1,17,017  (16.7%) | 8,507  (7.26%) | 1,59,691  (22.1%) | 7,315  (4.5%) | # | -2.7% |
| No | # | # | 5,82,668  (83.3%) | 30.638  (5.2%) | 5,62,423  (77.9%) | 19,814  (3.5%) | # | -1.7% |
| **Type of SLT** | **N, %** | **95% CI** | **N, %** | **95% CI** | **N, %** | **95% CI** |  |  |
| Gutkha/Paan masala | 2,267  (1.8%) | 1.7%-1.8% | 15,440  (2.2%) | (2.1%-2.3%) | 9,942  (1.3%) | 1.2%-1.4% | -0.4% | 0.9% |
| Khaini | Not Available | Not Available | 8,925  (1.2%) | (1.24%-1.3%) | 6,405  (0.8%) | 0.7%-0.9% | # | 0.4% |
| Paan with tobacco | 2,243  (1.8%) | 1.7%-1.9% | 12,780  (1.8% | (1.79%-1.9%) | 8,161  (1.1%) | 1%-1.2% | 0 | 0.7% |
| Chewing tobacco | 6,884  (5.53%) | 5.4%-5.6% | 4,643  (0.66%) | (0.64%-0.68%) | 2,382  (0.3%) | 0.2%-0.4% | -4.8% | 0.3% |
| Others | # | # | 1,029  (0.15%) | (0.13%-0.15%) | 2,509  (0.3%) | 0.3%-0.4% | # | 0.1% |

# Data not available


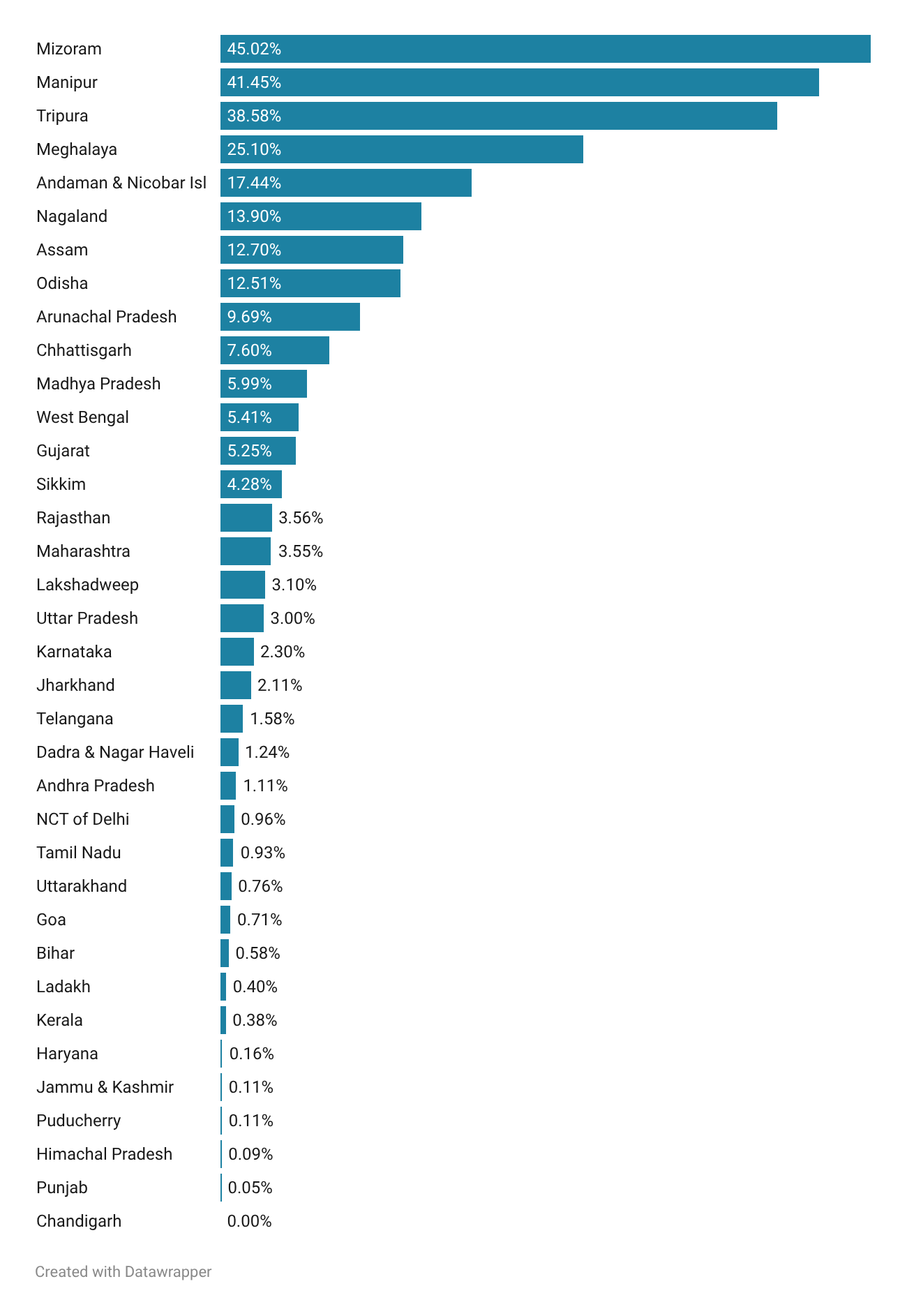
**Supplementary figure 1 :** Prevalence of smokeless tobacco use across Indian states
