# Supplementary material for: Trends and correlates of smokeless tobacco use among Indian women: Evidence from three rounds of the National Family Health Survey 2005–2021
Source: PLoS One. 2026 Aug 3;21(8):e0353704. doi: 10.1371/journal.pone.0353704 (PMC13432099; doi:10.1371/journal.pone.0353704)
Supplement: Attachment — Submitted filename: Author reply.docx [file pone.0353704.s005.docx]

**Editorial Comment**

This is an important and timely manuscript on smokeless tobacco use among Indian women, and it is generally well written with appropriate analysis. However, minor revisions are needed: strengthen the rationale for focusing on women of reproductive age, clarify handling of missing data and potential multicollinearity in the models, consider reporting model diagnostics, and provide 95% confidence intervals for prevalence estimates. Please also reconcile inconsistencies between descriptive and regression findings (e.g., rural–urban differences), expand discussion around key trends (such as rising urban use in NFHS-5), rephrase overly broad claims, and carefully revise language and formatting throughout

**Authors reply** : We thank the editor for the constructive feedback and for recognizing the importance of the manuscript. We have carefully revised the manuscript to address all the points raised. The introduction has been strengthened to better justify the focus on women of reproductive age. Details on handling missing data and assessment of multicollinearity have been clarified in the Methods section. Ninety five percent confidence intervals for prevalence estimates have been added where appropriate. We have also reviewed the descriptive and regression findings to ensure consistency and clarified the interpretation of rural urban differences. The discussion has been expanded to better contextualize key trends, including the increase in urban SLT use in the National Family Health Survey 5. Overly broad statements have been rephrased and the manuscript has been thoroughly edited to improve language, clarity, and formatting.

**Journal Comment**

Journal Requirements: When submitting your revision, we need you to address these additional requirements.

Please ensure that your manuscript meets PLOS ONE's style requirements, including those for file naming. The PLOS ONE style templates can be found at https://journals.plos.org/plosone/s/file?id=wjVg/PLOSOne_formatting_sample_main_body.pdf and <https://journals.plos.org/plosone/s/file?id=ba62/PLOSOne_formatting_sample_title_authors_affiliations.pdf>

**Authors reply**: We thank the editor for this reminder. The manuscript has been carefully revised to ensure compliance with PLOS ONE style and formatting requirements, including file naming. The manuscript has been updated.

2. We noticed you have some minor occurrence of overlapping text with the following previous publication(s), which needs to be addressed:- -doi: 10.1186/s12916-022-02662-0 In your revision ensure you cite all your sources (including your own works), and quote or rephrase any duplicated text outside the methods section. Further consideration is dependent on these concerns being addressed.

**Authors reply**: We thank the editor for bringing this to our attention. The manuscript has been carefully reviewed for overlapping text

3. Thank you for stating the following in the Competing Interests/Financial Disclosure section: The authors have declared that no competing interests exist. We note that one or more of the authors are employed by a commercial company: VART Consulting Pvt. Ltd a. Please provide an amended Funding Statement declaring this commercial affiliation, as well as a statement regarding the Role of Funders in your study. If the funding organization did not play a role in the study design, data collection and analysis, decision to publish, or preparation of the manuscript and only provided financial support in the form of authors' salaries and/or research materials, please review your statements relating to the author contributions, and ensure you have specifically and accurately indicated the role(s) that these authors had in your study. You can update author roles in the Author Contributions section of the online submission form. Please also include the following statement within your amended Funding Statement. “The funder provided support in the form of salaries for authors [insert relevant initials], but did not have any additional role in the study design, data collection and analysis, decision to publish, or preparation of the manuscript. The specific roles of these authors are articulated in the ‘author contributions’ section.” If your commercial affiliation did play a role in your study, please state and explain this role within your updated Funding Statement. b. Please also provide an updated Competing Interests Statement declaring this commercial affiliation along with any other relevant declarations relating to employment, consultancy, patents, products in development, or marketed products, etc. Within your Competing Interests Statement, please confirm that this commercial affiliation does not alter your adherence to all PLOS ONE policies on sharing data and materials by including the following statement: "This does not alter our adherence to PLOS ONE policies on sharing data and materials.” (as detailed online in our guide for authors http://journals.plos.org/plosone/s/competing-interests) . If this adherence statement is not accurate and there are restrictions on sharing of data and/or materials, please state these. Please note that we cannot proceed with consideration of your article until this information has been declared. Please include both an updated Funding Statement and Competing Interests Statement in your cover letter. We will change the online submission form on your behalf.

**Authors reply**: Thank you we have added this

4. We note that Figure 2 in your submission contain map images which may be copyrighted. All PLOS content is published under the Creative Commons Attribution License (CC BY 4.0), which means that the manuscript, images, and Supporting Information files will be freely available online, and any third party is permitted to access, download, copy, distribute, and use these materials in any way, even commercially, with proper attribution. For these reasons, we cannot publish previously copyrighted maps or satellite images created using proprietary data, such as Google software (Google Maps, Street View, and Earth). For more information, see our copyright guidelines: http://journals.plos.org/plosone/s/licenses-and-copyright. We require you to either (a) present written permission from the copyright holder to publish these figures specifically under the CC BY 4.0 license, or (b) remove the figures from your submission: a. You may seek permission from the original copyright holder of Figure 2 to publish the content specifically under the CC BY 4.0 license.  We recommend that you contact the original copyright holder with the Content Permission Form (http://journals.plos.org/plosone/s/file?id=7c09/content-permission-form.pdf) and the following text: “I request permission for the open-access journal PLOS ONE to publish XXX under the Creative Commons Attribution License (CCAL) CC BY 4.0 (http://creativecommons.org/licenses/by/4.0/). Please be aware that this license allows unrestricted use and distribution, even commercially, by third parties. Please reply and provide explicit written permission to publish XXX under a CC BY license and complete the attached form.” Please upload the completed Content Permission Form or other proof of granted permissions as an ""Other"" file with your submission. In the figure caption of the copyrighted figure, please include the following text: “Reprinted from [ref] under a CC BY license, with permission from [name of publisher], original copyright [original copyright year].” b. If you are unable to obtain permission from the original copyright holder to publish these figures under the CC BY 4.0 license or if the copyright holder’s requirements are incompatible with the CC BY 4.0 license, please either i) remove the figure or ii) supply a replacement figure that complies with the CC BY 4.0 license. Please check copyright information on all replacement figures and update the figure caption with source information. If applicable, please specify in the figure caption text when a figure is similar but not identical to the original image and is therefore for illustrative purposes only. The following resources for replacing copyrighted map figures may be helpful: USGS National Map Viewer (public domain): http://viewer.nationalmap.gov/viewer/ The Gateway to Astronaut Photography of Earth (public domain): http://eol.jsc.nasa.gov/sseop/clickmap/ Maps at the CIA (public domain): https://www.cia.gov/library/publications/the-world-factbook/index.html and https://www.cia.gov/library/publications/cia-maps-publications/index.html NASA Earth Observatory (public domain): http://earthobservatory.nasa.gov/ Landsat: http://landsat.visibleearth.nasa.gov/ USGS EROS (Earth Resources Observatory and Science (EROS) Center) (public domain): http://eros.usgs.gov/# Natural Earth (public domain): <http://www.naturalearthdata.com/>

**Authors reply:** We have removed the figure.

5. Please include captions for your Supporting Information files at the end of your manuscript, and update any in text citations to match accordingly. Please see our Supporting Information guidelines for more information: <http://journals.plos.org/plosone/s/supporting-information>.

**Authors reply**: We thank the editorial reviewer for this comment. Captions for all Supporting Information files have now been added at the end of the manuscript, and the corresponding in text citations have been updated to ensure consistency with the journal’s Supporting Information guidelines.

6. If the reviewer comments include a recommendation to cite specific previously published works, please review and evaluate these publications to determine whether they are relevant and should be cited. There is no requirement to cite these works unless the editor has indicated otherwise.

**Authors reply** : We thank the editorial reviewer for the suggestion.

7. Please review your reference list to ensure that it is complete and correct. If you have cited papers that have been retracted, please include the rationale for doing so in the manuscript text, or remove these references and replace them with relevant current references. Any changes to the reference list should be mentioned in the rebuttal letter that accompanies your revised manuscript. If you need to cite a retracted article, indicate the article’s retracted status in the References list and also include a citation and full reference for the retraction notice

**Authors reply** : We thank the editorial reviewer for this suggestion. The reference list has been carefully reviewed to ensure completeness and accuracy. No retracted articles were identified among the cited references. Minor corrections and updates have been made where necessary, and all changes to the reference list have been incorporated in the revised manuscript.

**Reviewer 1**

Dear Authors, thank you for a very interesting and important manuscript on smokeless tobacco use among women aged 15-49 years in India. The manuscript is generally well written and adds a valuable contribution to the ongoing tobacco control debates in India. I have a few comments that I believe will help enhance your work. I have provided them section by section for ease of reference.

**Introduction**

The introduction while provides a broader picture, I have the following observations which the authors may consider to address

i) It does not satisfactorily narrow down on why it’s important to look into the SLT use among women of reproductive age.
ii) Discussing on the existing gaps in tobacco control efforts and how women who use SLT, particularly those belonging to this age group are missed out might help to build the context.
iii) The specific independent variables and their inclusion is not adequately rationalized in the introduction section. As a suggestion it may be good to provide a conceptual framework given that the study is reliant on secondary analysis. Such a framework could help to justify the choice of selected factors associated.

**Author reply**

I. We thank the reviewer for the suggestion. The introduction already explains the relevance of studying smokeless tobacco among women of reproductive age by highlighting the burden of SLT use among women and its links with reproductive and maternal health outcomes. Since the analysis focuses on women aged 15 to 49 years as defined in the survey design, the current framing reflects the population under study. To avoid redundancy, we retained the structure while making minor clarifications to improve flow.
II. We agree that the introduction should better describe gaps in tobacco control efforts. We expanded the section to explain that smokeless tobacco often receives less policy attention compared with smoking and that women who use SLT, particularly in socio culturally accepted settings, remain under addressed in tobacco control programmes. This addition strengthens the policy context for examining SLT use among women.
III. We acknowledge the suggestion regarding a conceptual framework. The independent variables included in the analysis are standard socio demographic and household characteristics available in the NFHS dataset and commonly used in tobacco epidemiology studies using similar secondary survey data. These variables have been selected based on established literature examining determinants of tobacco use in India. Since the study relies on predefined variables from a nationally representative survey, a formal conceptual framework was not included.

**Methodology**

i) It was unclear how the researchers addressed missing data. Providing information about the same would be useful.
ii) There is a potential that several of independent variables could have multicollinearity and effect the results of multivariable models, did the authors test for it? Providing the details of the same would be valuable.
iii) Were any post-test estimates for regression models were computed (for example goodness of fit tests, sensitivity and specificity of models, and AUROC estimates)? If yes please provide them. If they were not done it might be good to check as large survey data have the issue of identifying highly significant associations but weak models.
iv) I assume that the authors have not pooled the samples from NFHS-3 to NFHS-5, and have descriptively reported the trend. While I am not advicing on pooling, I am curious to know if the authors have considered to do so as it has its advantages (eg. Assess the statistical significance of the changing trend in SLT prevalence).

**Authors Reply**

I. We thank the reviewer for raising this point. Observations with missing values in the variables used in the analysis were excluded during data preparation. The proportion of missing data was minimal and deletion did not affect the analytical sample or study findings. This clarification has been added to the methods section.
II. We agree that multicollinearity assessment is important in multivariable regression models. Variance Inflation Factor was examined for the independent variables included in the model. The VIF values were within acceptable limits, indicating no evidence of problematic multicollinearity.
III. We appreciate the reviewer’s suggestion regarding additional model diagnostics. The objective of the regression analysis in this study was to estimate associations between selected factors and SLT use using nationally representative survey data. Logistic regression with survey weights is widely used for such analyses in NFHS based studies. Therefore additional post estimation measures such as sensitivity, specificity, or AUROC were not undertaken.
IV. We thank the reviewer for this suggestion. The present analysis aimed to describe changes in prevalence and associated factors across survey rounds rather than estimate pooled effects. Therefore each NFHS round was analysed separately to preserve survey specific context and comparability across time. Pooling datasets was not undertaken as it was beyond the scope and objectives of the current analysis.

**Results**

i) Please provide 95% CI for the prevalence estimates in the text.
ii) The results from supplementary table does not seem to sync well with the results of multivariate table in some areas. With regard to the place of residence, rural regions had higher proportion of women using SLT in NFHS 3 & 4, whereas the odds seem to show that rural women had a lower probability of using SLT.

**Authors reply**

I. We thank the reviewer for this suggestion. The 95 % CI for prevalence estimates have now been added in the text where relevant to improve clarity and statistical reporting.
II. We appreciate the reviewer’s observation. The higher proportion of SLT use in rural areas in the descriptive table reflects unadjusted prevalence. The multivariable model estimates adjusted odds ratios after controlling for socio demographic factors such as age, education, wealth status, and region. After adjustment, the association between rural residence and SLT use changes direction, which explains the difference between descriptive proportions and adjusted estimates.

**Discussion**

i) Each of the findings could be better discussed. For example with reference to association between place of residence and SLT use. Its good that the researchers reported that the urban areas had higher SLT use during NFHS-5, but what could have led to this raise? Was it migration? COVID-19? Targeted SLT control interventions in states where it is highly prevalent ? discussion around this direction strengthen the arguments.
ii) The statements like ‘however the eastern part of country somehow managed to reduce the burden of SLT in last three rounds’ seems to be very loose. Rather discussing on what strategies could have contributed to this would be very helpful and might show a means for expansion to the regions where SLT persists to be high.

**Authors reply**

I. We thank the reviewer for this suggestion. The discussion has been expanded in the revised manuscript to provide possible explanations for the observed urban patterns in NFHS 5, including contextual factors and existing evidence.
II. We appreciate this comment. The revised discussion already elaborates on possible factors that contributed to the declining SLT burden in the eastern region and highlights implications for other regions.

**Methodological limitation**

i) “to our knowledge, this is the first study that contributes to understanding the trends of SLT usage among women using the latest NFHS surveys”. I have come across the studies that have done similar work based on NFHS surveys. It might be good to remove/reword this statement as it may not add much.
ii) To the limitation it may be better to add the context of social desirability bias, specifically given that the NFHS-5 has been conducted during the COVID-19 years, chewing tobacco use could have been looked on from a stigmatised lens so people may not have reported even if they consumed.

**Authors reply:**

I. We agree and have reworded the statement to avoid claiming novelty.
II. We thank the reviewer for this suggestion. We have incorporated social desirability bias and the COVID 19 survey context as additional limitations in the revised manuscript.

**General comment**

The writing can be improved. Please carefully check the grammar, formatting, font differences and typographic errors in the manuscript.

**Authors reply** : We thank the reviewer for this observation. The manuscript has been carefully revised to improve language clarity, grammar, formatting consistency, and typographic errors. The entire document has been thoroughly proofread and corrected in the revised version.

**Reviewer 2**

Dear Authors,

Thank you for the opportunity to review your manuscript. It’s really an important piece of work considering the neglected oral health worldwide and practice of poor oral health awareness practice specially in Asian countries.

I am offering some gentle suggestions that is required. The goal is not to critique for the sake of critique, but to help elevate a promising script into one that can speak more clearly and powerfully to readers.

Thank you

**Recommendations**

**Abstract**

**Line 51 -55 :** Result section lacks important findings and numeric estimates. Reporting sample size is okay, you must add estimates for other important findings such as odds ratios with 95% CIs. This part needs to be rewritten.

**Authors Reply**: Thank you for your suggestion we have now improved the result section of the Abstract.

**Line 53:** “Gutkha/ Paan masala was the most commonly used….” Reported percentage would be more engaging. So you should consider reporting percentage

**Authors Reply:** Thank you for your suggestion we have now improved the result section of the Abstract.

**Line 54:** “Mizoram had the highest (45%) prevalence of SLT use.” This statement is confusing. As the each phase of survey analyzed separately, readers will ask, in which phase did Mizoram have highest prevelance? Was it NFHS 3/4/5?? So this line must be clarified

**Authors Reply** Thank you for your suggestion we have now improved the result section of the Abstract.

**Line 54-55:** You must report aOR with 95% CI for the determinants so that readers can easily understand the impact of those determinants.

Also, as the three phases of survey analyzed separately, reporting the key findings and determinants from each survey will strengthen the core message of the manuscript. Include key determinants with direction (e.g., lower education, older age) Consider this and incorporate it into the abstract, at least the results.

**Authors Reply: Thank you for your suggestion**

**Line 50-60:** ‘…..urgent need to strengthen efforts to prevent the initiation of smokeless tobacco (SLT) use and to support its cessation…..’ seems confusing. As readers read that SLT use has declined from NFHS 3 – 5, aren’t there any prevention or cessation strategy already implemented? You should consider paraphrasing this recommendation accordingly. For example: If there isn’t any program to prevent or support cessation of SLT use, mention it and then recommend. If there is, consider the shortcomings/lackings of those programs and recommend suggestions accordingly. That will strengthen the value of this work as well.

Overall, The abstract seems to lack information given that the study included huge number of study population.

**Authors Reply: done**

**Introduction**

**Line 73-76:** Mention reference(s) of the evidence.

Done already

**Line 115-116 and 122:** “….smoked tobacco in form of cigarettes….” were categorized as ‘SLT Non user’ in Outcome variable. Then again ‘cigarette/tobacco smoking’ is considered as independent variable. How do these to differ? Needs clarification.

**Authors Reply: Thank you for your suggestion**

**Line 126-127:** Needs proper reference/justification of keeping non-religious participants with other religious groups

**Authors Reply: Thank you, this is based on previous classifications in literatures**

**Line 135-136:** job type ‘clerical’ is kept in multiple categories. Must clarify this.

**Authors Reply: Thank you, corrected**

**Line 142-143:** Though BMI calculation formula for teen ages is the same, but interpretation greatly differs, especially for teen female. For them, it isn’t categorized by specific cut-off points, rather it is categorized by sex specific BMI for age percentiles. (Ref: [Child and Teen BMI Categories | BMI | CDC](https://www.cdc.gov/bmi/child-teen-calculator/bmi-categories.html#:~:text=BMI%20categories%20for%20children%20and,used%20in%20calculating%20BMI%20percentiles.)). So for Under 20 females, BMI category might be mis-specified, please recalculate for that specific age group.

**Authors Reply:** We thank the reviewer for this important observation. NFHS surveys report nutritional status for women aged 15 to 49 years using standard adult BMI cut offs. This approach is followed in national reports coordinated by the International Institute for Population Sciences and in analyses based on the Demographic and Health Surveys program. Several peer reviewed studies using NFHS data have applied uniform adult BMI categories across the full 15 to 49 year age range to ensure consistency and comparability across survey rounds.

Ref 1: <https://link.springer.com/article/10.1186/s12889-025-21635-5>

Ref 2 : <https://link.springer.com/article/10.1186/s12905-022-01786-y>

Ref 3: <https://pmc.ncbi.nlm.nih.gov/articles/PMC10611613/>

**Line 156-157:** ‘The statistically significant variables (p<0.05) from the unadjusted model were used to run a multivariate logistic regression…..’ needs reference for opting this approach. stepwise screening by p-value is not recommended in modern epidemiology, It is expected to select covariates based on conceptual frameworks (DAG/causal reasoning) and prior literature, not significance screening. Epidemiologists also utilize variables with p value <0.251 in the bivariate chi-square table to fit the model as well. I am not saying the approach you followed is wrong, but you need to add reference that this approach is also considerable.

**Authors reply:**

We thank the reviewer for this important methodological comment. We have now clarified in the Methods section that variable selection was informed by prior literature and epidemiologic relevance, and that the univariable screening step followed established purposeful selection approaches described in the above references.

**Line 157:** the words ‘multivariate’ and ‘multivariable’ logistic regression are not the same. From this context, I think you employed ‘multivariable logistic regression’. Please correct.

**Authors reply:** Thanks for pointing out corrected

**Results**

**Line 165-166:** As you mentioned that you categorized the age group, mentioning the mean age is unnecessary and adds little to no significance to the result.

**Authors reply:** While age was categorized for regression analysis, we feel reporting the mean age serves a descriptive purpose and helps readers understand the overall age structure of the study population across survey rounds. Since our analysis spans three NFHS waves conducted over a 15 year period, presenting the mean age provides context on whether the age distribution of women 15 to 49 years shifted over time. We understand, descriptive statistics such as mean age are routinely reported in population based studies, even when age is modeled categorically, to allow comparison with other studies and to document temporal changes in the sample composition. In our case, the gradual increase in mean age across rounds reflects demographic shifts in the surveyed population, which is relevant for interpreting trends in smokeless tobacco use.

**Line 168:** Descriptive table is one of the core components of any quantitative manuscript. Instead of supplementary table, you must provide the descriptive table in the manuscript it.

Your manuscript lacks a descriptive table of the study population and bivariate (χ²) analyses examining the association between smokeless tobacco use and socio-demographic variables. This omission limits transparency and interpretability of the regression findings. For a large-scale survey-based epidemiological study, Q1 journals typically expect weighted descriptive statistics and survey-adjusted bivariate analyses prior to multivariable modelling. You should include (i) a descriptive table stratified by SLT use status and NFHS round, and (ii) design-adjusted χ² tests for bivariate associations, clearly stating the survey procedures used.

**Authors reply:** We thank the reviewer for this constructive suggestion.

We agree that descriptive statistics are central to transparency in epidemiological research. In the current manuscript, the descriptive characteristics of the study population across NFHS rounds are presented in detail in the Supplementary table to maintain readability and adhere to word and table limits. Given the inclusion of multiple covariates across three survey rounds, inclusion of the full weighted descriptive table in the main manuscript would substantially increase its length and reduce clarity of the primary findings.

**Line 173-174:** Without the descriptive table, this type of claim sounds incomplete, erratic and discordant, doesn’t make any sense. Need to write these more explicitly describing relevant information. (e.g. ‘whereas Gutkha/ Paan masala and Paan with tobacco (1.8%) were utilized in a similar manner” what do you mean by similar manner? Are the percentage of users same for Gutkha and Paan with tobacco? Then the report must include both percentage. Gutkha/ Paan masala (1.8%) and Paan with tobacco (1.8%) were utilized in a similar manner.

**Authors reply:** We thank the reviewer for pointing this out. We have now revised the sentence to report the exact percentages for both products.

The revised text reads: “During NFHS 3, chewing tobacco was the most commonly used product at 5.5 percent, while Gutkha or Paan masala at 1.8 percent and Paan with tobacco at 1.8 percent showed equal prevalence.”

**Line 180** – in text citation of Figures and tables must be added in the paragraph just prior to the figures/tables. Please follow the figure formatting guideline: [Figures | PLOS One](https://journals.plos.org/plosone/s/figures)

**Authors reply:** We thank the reviewer for highlighting this formatting issue. We have revised the manuscript to ensure that all figures and tables are cited in the text immediately before their first appearance, in accordance with journal formatting guidelines.

**Line 186:** “SLT use among women is **significantly** lower in NFHS-5…..” claim is abrupt and has no statistical backings. What statistical method did you utilize to assess the significance decline? You did not mention that in methods. Proportional significance can be obtained from Chi-square test of goodness of fit or test of independence. As all of your variables are categorical, utilizing this would give the result statistical basis to call it significant decline. You should consider running this test for the variables and then state ‘significant proportional change’

**Authors reply:** We thank the reviewer for this observation. We agree that the term “significant” requires an explicitly stated statistical test and corresponding description in the Methods section. To avoid overstating, we have removed the word “significant” from the sentence describing the decline in SLT use across NFHS rounds.

**Line 205** – same comment as for line 186. You need to run proper statistical method before concluding significant decrease!

**Authors reply:** Thank you for pointing out, corrected

**Line 208 – 212** – aORs are collapsible and from three different models; are not directly comparable because the baseline odds, variance structure, heterogeneity and most importantly sample composition differs in all three models. You cannot infer a difference between coefficients just by eyeballing them across separate models. It’s a classic statistical error.

Correct way to infer is to use pooled regression model with interaction terms in a dataset where all the observation from all three survey rounds are combined. The equation is provided below:

$$Logit\left( Y \right)=X^{*}+X_{n}+Round+(X^{*}\times Round)$$

Here,

Y = your outcome which is usage of SLT

X^*^ = Desired Determinants (for example, Age group)

X_n_ = Other correlates

Round = A variable specifying observation from each round (NFHS 3, 4 or 5)

X^*^ x Round = Interaction term of Age group and round. Estimate of which will give the aOR changes across years within age groups, and then you can claim the aOR change.

For other variables of interest, just change the desired determinant accordingly to get comparable change in aOR. Just replace age group with that variable in the interaction term.

Also, model specification and fit assessment is necessary for declaring determinants confidently.so reporting these statistics is mandatory. You have mentioned taking survey weights into account when you analyzed, but did you consider other survey-design elements? For example, PSUs, survey strata? Also as survey sampling method usually follows multistage hierarchical approach, so a multilevel regression model is also more appropriate for survey data. Though utilizing these into analysis is not mandatory, yet you have to clarify that too into the manuscript like clearly stating that these elements were not taken into consideration and what the implications are for not taking those into consideration, so that readers are full aware of the implications. So, you must either re-analyze or acknowledge this limitation properly.

**Author reply:** We thank the reviewer for this detailed methodological comment. We agree that adjusted odds ratios derived from three separate models should not be directly compared by visual inspection, as differences in baseline odds, sample composition, and variance structure across survey rounds may affect estimates. We also acknowledge that a pooled regression model with interaction terms between survey round and key determinants would provide a more rigorous framework to formally test changes in associations over time. In the current manuscript, our intent was descriptive, to present determinants within each NFHS round rather than to statistically test differences in coefficients across rounds. We did not formally estimate interaction terms in a pooled model, and therefore we have revised the text to avoid language that implies direct statistical comparison of adjusted odds ratios across surveys.

**So these 208-212** lines are statistically invalid. Please make correction if you really want to report the change in odds ratio over the rounds of survey. If not, then you must report the aORs separately for three different models without comparing aORs from each round.

**Author reply**: We thank the reviewer for this important clarification. We agree that direct comparison of adjusted odds ratios across three independently estimated models is statistically inappropriate without formal interaction testing in a pooled framework.

**Line 219** – again, you can’t claim ‘significant rise’ by comparing aOR between rounds, must include interaction term in the model as commented before. “significant rise in the risk of SLT usage”, binomial logistic model doesn’t give the Risk Ratio that you can state. Odds Ratio and Risk Ratio cannot be used interchangeably.

Author reply: We thank the reviewer for this important clarification, revised the texts

**Line 220** – Don’t use subjective words like ‘considerable’ (or comparable/reasonable/notable/ large, small or moderate change) without statistical evidence. For You can report p value, effect size, or magnitude of change in % then claim the words. Again, aORs are not appropriate to declare ‘considerable drop’ by eyeballing without statistical tests.

**Author reply**: Thank you for highlighting, we have now revised the wordings

**Line 225-227** – Please also mention the estimates with confidence interval as well. Those are the things that readers expect to see in the result section.

**Authors reply**: Revised this, thank you

**Line 235** – You can not use ‘Multivariate’ and ‘Multivariable’ interchangeably

Please follow standard reporting of odds ratios in paragraphs and tables as well. Following literature would help: [How to Clearly and Accurately Report Odds Ratio and Hazard Ratio in Diagnostic Research Studies? - PMC](https://pmc.ncbi.nlm.nih.gov/articles/PMC9340231/), [Requiem for odds ratios - Norton - 2024 - Health Services Research - Wiley Online Library](https://onlinelibrary.wiley.com/doi/full/10.1111/1475-6773.14337?msockid=0be00de0ebd467ab39931b83ea4f66cf)

**Authors reply**: Thank you, we have now revised the error.

**Table 01 –**

1. You should clarify why ‘Others’ group was considered as reference groups for some variables

2. Please keep the reference category of all variables in the first row where variable is placed. For example, Wealth index should be rearranged like this: Richest (ref), Richer, Middle, Poorer, Poorest. Follow the table formatting guideline: [Tables | PLOS One](https://journals.plos.org/plosone/s/tables)

**Authors reply:**

1. The category “Others” was selected as the reference group to allow meaningful comparison with the largest and most heterogeneous segment in the dataset. This approach ensured model stability and adequate sample size within the reference category.

2. We understand the suggestion to place the reference category in the first row for each variable. However, we have retained the current ordering to maintain consistency and uniformity with the supplementary tables and previously defined category structures. Rearranging the categories would alter alignment across tables and affect comparability. For clarity, we have clearly marked the reference category in the table already.

**Line 238 – 245** This part of the manuscript seems to be misplaced. Please follow a cohesion and strict and consistent reporting pattern throughout the manuscript. The general reporting format for result section is given below:

1. Report descriptive tables first

2. then report bivariate table (if any)

3. then report regression tables.

In these lines, you reported the the frequency percentage across different states. This is a part of descriptive table, which you also reported earlier, so after reporting regression table, placement of this paragraph is abrupt. Please report this section earlier in the descriptive section.

State-wise prevalence (NFHS-5) reporting needs precision as well. You reported Chandigarh 0%, Punjab 0.05%, Himachal 0.09%. Given sampling, true zero is unlikely, is it could be rounding or small sample? Provide CIs and sample sizes by state. Without CIs, readers may misinterpret. So add a state-level table (supplement) with weighted prevalence, 95% CI, and n. Avoid “0%” unless n is extremely small and CI reported.

**Author reply**: Thank you for your careful review and suggestion regarding the reporting structure. We agree with the recommended order for presenting results. Accordingly, we have revised the manuscript to ensure a consistent and logical flow. All descriptive findings, including the frequency and percentage distribution across states, are now reported in the descriptive section before presenting the regression results.

Thank you for this important observation. We agree that reporting “0%” without precision measures may lead to misinterpretation, particularly given sampling variability. The observed values were influenced by small sample sizes and rounding. In response, we have added a supplementary state level table that reports weighted prevalence along with 95% confidence intervals and corresponding sample sizes for each state. We have avoided reporting “0%” and now present estimates with appropriate precision.

**Line 245:** Please follow the figure formatting guideline of PLoS: [Figures | PLOS One](https://journals.plos.org/plosone/s/figures). For this figure, put special attention to the ‘Maps’ subsection of this link.

**Authors reply:** Thank you noted but we have now, removed the heat map image instead presented the findings in the bar chart

**Discussion**

**Line 256** – ‘….and associated risk factors…..’, you can not call these risk factors unless there is proven causality assumption. ‘Determinants/factors associated with…’ will be appropriate phrasing.

**Authors reply**: Thank you this is done.

Discussion section should play more role to explaining the different findings with more focus on how, why rather than just repeating the results. For example

**Line 260-261** **–** ‘however some of the previous Indian studies reported higher prevalence of SLT use.’ Discussion must include how and why those studies reported higher prevalences, and what could be the reasons that your study findings differ from those.

**Authors reply**: Thank, we have revised this section.

Line -266 – “tobacco usage increased with age” is interesting finding, but again why women as their age increase, tend to use SLT would be more impactful for intervention design. So if you find findings from literature that explain why is it so, it would add great value to your work.

Same comment for **Line 269-271,**

Authors reply : Thank you for your comment, we have edited this part.

**Line 281:** You suddenly talked about “Hypertensive SLT users”, but hypertension is not described in Methods and not shown in Results. Sudden claim like this are abrupt and not appropriate at the discussion section. If you think it’s key finding, please describe about it in the result section, as well as the variables section where you described each variable.

**Authors reply**: Thank you, we have now corrected this part.

**Line 282-284:** Repetitive information. Reported on line 267. These lines can be place together if you think it’s different

**Authors reply**: Thank you, we have now corrected this part.

**Line 296-297:** Repetitive information. Reported on line 277

**Authors reply**: Thank you, we have now corrected this part.

**Line 300 -301:** if you would add how that part of country managed to reduce the burden, that can be one of your core recommendations for all other parts. I strongly suggest to follow my previous comments for this section.

Author reply: Thank you for your recommendations.

**Line 306**: ‘influences women's use of SLT through peer effects.’ What is the basis of this claim? Your study didn’t find that. If it is from any literature, you must add reference.

**Author reply**: Thank you, we have now edited this and added suitable references .

Overall, the discussion section lacks in-depth exploration and explanation. This section needs to be rewritten and well referenced backings for the determinants. Please revise accordingly

**Authors reply**: Thank you we have edited this and we think this has been improved substantially.

**Policy Implications**

**Line 324 – 326;** Needs reference

**Line 327 – 342**: this sort of claim should be placed in recommendation section.

Policy implication and Recommendation serve different purposes in a manuscript. Policy implications explain what the findings mean for existing or potential strategies, without prescribing specific actions that is for recommendations.

Also for the recommendations, these strong throw of words must need literature support and prior empirical evidence, either within the country or in other countries. So these recommendations must be backed by references. You must add references for those, may what other countries have done to reduce the burden of SLT. If there isn’t any empirical evidence, you must adopt a cautious and prospective tone**,** use conditional language (e.g., **may*,* could*,* has the potential to**) for recommendations, clearly acknowledge the lack of empirical evidence, and position these recommendations as a future policy research or direction grounded in theory or related evidence.

**Author reply** : We thank the reviewer for this important clarification regarding the distinction between policy implications and recommendations. We agree that policy implications should interpret the meaning of the findings for current strategies, whereas recommendations should propose specific actions supported by empirical evidence.

**Verdict: Major Revision Needed**

The topic is important and policy-relevant. Using three NFHS rounds to examine temporal changes and determinants of smokeless tobacco (SLT) use among women is a strength, as is the large sample and the attempt to map state variation. However, several core methodological details are missing or unclear (survey design handling, outcome comparability across rounds, model specification, missing data, and interpretation). The writing also needs tightening, and some claims in Discussion/Policy implications require stronger evidence or careful rephrasing.

You broadly need to work on

1) Outcome definition and cross-round comparability

2) Survey design and weighting and Analysis

4) Interpretation issues and claims

5) Presentation of results: descriptive and regression table readability, formatting as per journal guideline

6) Figure formatting as per journal guideline

6) Support of Claims in Discussion/Policy implications:

7) Separate Recommendation section

Add frequency tables and bivariate tables for each round just like you reported regression table. Revising the whole analysis would be a hassle, so I suggest you keep the regression analysis as it is. But you must revise the interpretations without cross-round comparison of aORs, write the findings individually for each round. And state “not incorporating the survey strata or PSUs in the analysis and it’s implications’ in the methodological limitation section. Please incorporate the suggestions accordingly.

I appreciate the hard works you put on this work.

Warm Regards.

**Author reply**: We thank the reviewer for the detailed review and constructive suggestions. We revised the manuscript accordingly and believe these changes improved the clarity, methodological rigor, and overall quality of the manuscript.
