# Supplementary material for: Trends and correlates of smokeless tobacco use among Indian women: Evidence from three rounds of the National Family Health Survey 2005–2021
Source: PLoS One. 2026 Aug 3;21(8):e0353704. doi: 10.1371/journal.pone.0353704 (PMC13432099; doi:10.1371/journal.pone.0353704)
Supplement: Attachment — Submitted filename: Review Comments for PLOS SLT article.docx [file pone.0353704.s002.docx]

Dear Authors, thank you for a very interesting and important manuscript on smokeless tobacco use among women aged 15-49 years in India. The manuscript is generally well written and adds a valuable contribution to the ongoing tobacco control debates in India. I have a few comments that I believe will help enhance your work. I have provided them section by section for ease of reference.

**Introduction**

The introduction while provides a broader picture, I have the following observations which the authors may consider to address

i) It does not satisfactorily narrow down on why it’s important to look into the SLT use among women of reproductive age.
ii) Discussing on the existing gaps in tobacco control efforts and how women who use SLT, particularly those belonging to this age group are missed out might help to build the context.
iii) The specific independent variables and their inclusion is not adequately rationalized in the introduction section. As a suggestion it may be good to provide a conceptual framework given that the study is reliant on secondary analysis. Such a framework could help to justify the choice of selected factors associated.

**Methodology**

i) It was unclear how the researchers addressed missing data. Providing information about the same would be useful.
ii) There is a potential that several of independent variables could have multicollinearity and effect the results of multivariable models, did the authors test for it? Providing the details of the same would be valuable.
iii) Were any post-test estimates for regression models were computed (for example goodness of fit tests, sensitivity and specificity of models, and AUROC estimates)? If yes please provide them. If they were not done it might be good to check as large survey data have the issue of identifying highly significant associations but weak models.
iv) I assume that the authors have not pooled the samples from NFHS-3 to NFHS-5, and have descriptively reported the trend. While I am not advicing on pooling, I am curious to know if the authors have considered to do so as it has its advantages (eg. Assess the statistical significance of the changing trend in SLT prevalence).

**Results**

i) Please provide 95% CI for the prevalence estimates in the text.
ii) The results from supplementary table does not seem to sync well with the results of multivariate table in some areas. With regard to the place of residence, rural regions had higher proportion of women using SLT in NFHS 3 & 4, whereas the odds seem to show that rural women had a lower probability of using SLT.

**Discussion**

i) Each of the findings could be better discussed. For example with reference to association between place of residence and SLT use. Its good that the researchers reported that the urban areas had higher SLT use during NFHS-5, but what could have led to this raise? Was it migration? COVID-19? Targeted SLT control interventions in states where it is highly prevalent ? discussion around this direction strengthen the arguments.
ii) The statements like ‘however the eastern part of country somehow managed to reduce the burden of SLT in last three rounds’ seems to be very loose. Rather discussing on what strategies could have contributed to this would be very helpful and might show a means for expansion to the regions where SLT persists to be high.

**Methodological limitation**

i) “to our knowledge, this is the first study that contributes to understanding the trends of SLT usage among women using the latest NFHS surveys”. I have come across the studies that have done similar work based on NFHS surveys. It might be good to remove/reword this statement as it may not add much.
ii) To the limitation it may be better to add the context of social desirability bias, specifically given that the NFHS-5 has been conducted during the COVID-19 years, chewing tobacco use could have been looked on from a stigmatised lens so people may not have reported even if they consumed.

**General comment**

The writing can be improved. Please carefully check the grammar, formatting, font differences and typographic errors in the manuscript.
