# Supplementary material for: Trends and correlates of smokeless tobacco use among Indian women: Evidence from three rounds of the National Family Health Survey 2005–2021
Source: PLoS One. 2026 Aug 3;21(8):e0353704. doi: 10.1371/journal.pone.0353704 (PMC13432099; doi:10.1371/journal.pone.0353704)
Supplement: Attachment — Submitted filename: Review on PONE-D-25-25520.docx [file pone.0353704.s003.docx]

Dear Authors,

Thank you for the opportunity to review your manuscript. It’s really an important piece of work considering the neglected oral health worldwide and practice of poor oral health awareness practice specially in Asian countries.

I am offering some gentle suggestions that is required. The goal is not to critique for the sake of critique, but to help elevate a promising script into one that can speak more clearly and powerfully to readers.

Thank you

# Recommendations

**Abstract**

**Line 51 -55 :** Result section lacks important findings and numeric estimates. Reporting sample size is okay, you must add estimates for other important findings such as odds ratios with 95% CIs. This part needs to be rewritten.

**Line 53:** “Gutkha/ Paan masala was the most commonly used….” Reported percentage would be more engaging. So you should consider reporting percentage

**Line 54:** “Mizoram had the highest (45%) prevalence of SLT use.” This statement is confusing. As the each phase of survey analyzed separately, readers will ask, in which phase did Mizoram have highest prevelance? Was it NFHS 3/4/5?? So this line must be clarified

**Line 54-55:** You must report aOR with 95% CI for the determinants so that readers can easily understand the impact of those determinants.

Also, as the three phases of survey analyzed separately, reporting the key findings and determinants from each survey will strengthen the core message of the manuscript. Include key determinants with direction (e.g., lower education, older age) Consider this and incorporate it into the abstract, at least the results.

**Line 50-60:** ‘…..urgent need to strengthen efforts to prevent the initiation of smokeless tobacco (SLT) use and to support its cessation…..’ seems confusing. As readers read that SLT use has declined from NFHS 3 – 5, aren’t there any prevention or cessation strategy already implemented? You should consider paraphrasing this recommendation accordingly. For example: If there isn’t any program to prevent or support cessation of SLT use, mention it and then recommend. If there is, consider the shortcomings/lackings of those programs and recommend suggestions accordingly. That will strengthen the value of this work as well.

Overall, The abstract seems to lack information given that the study included huge number of study population.

**Introduction**

**Line 73-76:** Mention reference(s) of the evidence.

**Line 115-116 and 122:** “….smoked tobacco in form of cigarettes….” were categorized as ‘SLT Non user’ in Outcome variable. Then again ‘cigarette/tobacco smoking’ is considered as independent variable. How do these to differ? Needs clarification.

**Line 126-127:** Needs proper reference/justification of keeping non-religious participants with other religious groups

**Line 135-136:** job type ‘clerical’ is kept in multiple categories. Must clarify this.

**Line 142-143:** Though BMI calculation formula for teen ages is the same, but interpretation greatly differs, especially for teen female. For them, it isn’t categorized by specific cut-off points, rather it is categorized by sex specific BMI for age percentiles. (Ref: [Child and Teen BMI Categories | BMI | CDC](https://www.cdc.gov/bmi/child-teen-calculator/bmi-categories.html#:~:text=BMI%20categories%20for%20children%20and,used%20in%20calculating%20BMI%20percentiles.)). So for Under 20 females, BMI category might be mis-specified, please recalculate for that specific age group.

**Line 156-157:** ‘The statistically significant variables (p<0.05) from the unadjusted model were used to run a multivariate logistic regression…..’ needs reference for opting this approach. **stepwise screening by p-value** is not recommended in modern epidemiology, It is expected to select covariates based on conceptual frameworks (DAG/causal reasoning) and prior literature, not significance screening. Epidemiologists also utilize variables with p value <0.251 in the bivariate chi-square table to fit the model as well. I am not saying the approach you followed is wrong, but you need to add reference that this approach is also considerable.

**Line 157:** the words ‘multivariate’ and ‘multivariable’ logistic regression are not the same. From this context, I think you employed ‘multivariable logistic regression’. Please correct.

**Results**

**Line 165-166:** As you mentioned that you categorized the age group, mentioning the mean age is unnecessary and adds little to no significance to the result.

**Line 168:** Descriptive table is one of the core components of any quantitative manuscript. Instead of supplementary table, you must provide the descriptive table in the manuscript it.

Your manuscript lacks a descriptive table of the study population and bivariate (χ²) analyses examining the association between smokeless tobacco use and socio-demographic variables. This omission limits transparency and interpretability of the regression findings. For a large-scale survey-based epidemiological study, Q1 journals typically expect weighted descriptive statistics and survey-adjusted bivariate analyses prior to multivariable modelling. You should include (i) a descriptive table stratified by SLT use status and NFHS round, and (ii) design-adjusted χ² tests for bivariate associations, clearly stating the survey procedures used.

**Line 173-174:** Without the descriptive table, this type of claim sounds incomplete, erratic and discordant, doesn’t make any sense. Need to write these more explicitly describing relevant information. (e.g. ‘whereas Gutkha/ Paan masala and Paan with tobacco (1.8%) were utilized in a similar manner” what do you mean by similar manner? Are the percentage of users same for Gutkha and Paan with tobacco? Then the report must include both percentage. Gutkha/ Paan masala (1.8%) and Paan with tobacco (1.8%) were utilized in a similar manner.

**Line 180** – in text citation of Figures and tables must be added in the paragraph just prior to the figures/tables. Please follow the figure formatting guideline: [Figures | PLOS One](https://journals.plos.org/plosone/s/figures)

**Line 186:** “SLT use among women is **significantly** lower in NFHS-5…..” claim is abrupt and has no statistical backings. What statistical method did you utilize to assess the significance decline? You did not mention that in methods. Proportional significance can be obtained from Chi-square test of goodness of fit or test of independence. As all of your variables are categorical, utilizing this would give the result statistical basis to call it significant decline. You should consider running this test for the variables and then state ‘significant proportional change’

**Line 205** – same comment as for line 186. You need to run proper statistical method before concluding significant decrease!

**Line 208 – 212** – aORs are collapsible and from three different models; are not directly comparable because the baseline odds, variance structure, heterogeneity and most importantly sample composition differs in all three models. You cannot infer a difference between coefficients just by eyeballing them across separate models. It’s a classic statistical error.

Correct way to infer is to use pooled regression model with interaction terms in a dataset where all the observation from all three survey rounds are combined. The equation is provided below:

$$Logit\left( Y \right)=X^{*}+X_{n}+Round+(X^{*}\times Round)$$

Here,

Y = your outcome which is usage of SLT

X^*^ = Desired Determinants (for example, Age group)

X_n_ = Other correlates

Round = A variable specifying observation from each round (NFHS 3, 4 or 5)

X^*^ x Round = Interaction term of Age group and round. Estimate of which will give the **aOR changes across years** within age groups, and then you can claim the aOR change.

For other variables of interest, just change the desired determinant accordingly to get comparable change in aOR. Just replace age group with that variable in the interaction term.

Also, model specification and fit assessment is necessary for declaring determinants confidently.so reporting these statistics is mandatory. You have mentioned taking survey weights into account when you analyzed, but did you consider other survey-design elements? For example, PSUs, survey strata? Also as survey sampling method usually follows multistage hierarchical approach, so a multilevel regression model is also more appropriate for survey data. Though utilizing these into analysis is not mandatory, yet you have to clarify that too into the manuscript like clearly stating that these elements were not taken into consideration and what the implications are for not taking those into consideration, so that readers are full aware of the implications. So, you must either re-analyze or acknowledge this limitation properly.

**So these 208-212** lines are statistically invalid. Please make correction if you really want to report the change in odds ratio over the rounds of survey. If not, then you must report the aORs separately for three different models without comparing aORs from each round.

**Line 219** – again, you can’t claim ‘significant rise’ by comparing aOR between rounds, must include interaction term in the model as commented before. “significant rise in the risk of SLT usage”, binomial logistic model doesn’t give the Risk Ratio that you can state. Odds Ratio and Risk Ratio cannot be used interchangeably.

**Line 220** – Don’t use subjective words like ‘considerable’ (or comparable/reasonable/notable/ large, small or moderate change) without statistical evidence. For You can report p value, effect size, or magnitude of change in % then claim the words. Again, aORs are not appropriate to declare ‘considerable drop’ by eyeballing without statistical tests.

**Line 225-227** – Please also mention the estimates with confidence interval as well. Those are the things that readers expect to see in the result section.

**Line 235** – You can not use ‘Multivariate’ and ‘Multivariable’ interchangeably

Please follow standard reporting of odds ratios in paragraphs and tables as well. Following literature would help: [How to Clearly and Accurately Report Odds Ratio and Hazard Ratio in Diagnostic Research Studies? - PMC](https://pmc.ncbi.nlm.nih.gov/articles/PMC9340231/), [Requiem for odds ratios - Norton - 2024 - Health Services Research - Wiley Online Library](https://onlinelibrary.wiley.com/doi/full/10.1111/1475-6773.14337?msockid=0be00de0ebd467ab39931b83ea4f66cf)

**Table 01 –**

1. You should clarify why ‘Others’ group was considered as reference groups for some variables

2. Please keep the reference category of all variables in the first row where variable is placed. For example, Wealth index should be rearranged like this: Richest (ref), Richer, Middle, Poorer, Poorest. Follow the table formatting guideline: [Tables | PLOS One](https://journals.plos.org/plosone/s/tables)

**Line 238 – 245** This part of the manuscript seems to be misplaced. Please follow a cohesion and strict and consistent reporting pattern throughout the manuscript. The general reporting format for result section is given below:

1. Report descriptive tables first

2. then report bivariate table (if any)

3. then report regression tables.

In these lines, you reported the the frequency percentage across different states. This is a part of descriptive table, which you also reported earlier, so after reporting regression table, placement of this paragraph is abrupt. Please report this section earlier in the descriptive section.

State-wise prevalence (NFHS-5) reporting needs precision as well. You reported Chandigarh 0%, Punjab 0.05%, Himachal 0.09%. Given sampling, true zero is unlikely, is it could be rounding or small sample? Provide CIs and sample sizes by state. Without CIs, readers may misinterpret. So add a state-level table (supplement) with weighted prevalence, 95% CI, and n. Avoid “0%” unless n is extremely small and CI reported.

**Line 245:** Please follow the figure formatting guideline of PLoS: [Figures | PLOS One](https://journals.plos.org/plosone/s/figures). For this figure, put special attention to the ‘Maps’ subsection of this link.

**Discussion**

**Line 256** – ‘….and associated risk factors…..’, you can not call these risk factors unless there is proven causality assumption. ‘Determinants/factors associated with…’ will be appropriate phrasing.

Discussion section should play more role to explaining the different findings with more focus on how, why rather than just repeating the results. For example

**Line 260-261** **–** ‘however some of the previous Indian studies reported higher prevalence of SLT use.’ Discussion must include how and why those studies reported higher prevalences, and what could be the reasons that your study findings differ from those.

Line -266 – “tobacco usage increased with age” is interesting finding, but again why women as their age increase, tend to use SLT would be more impactful for intervention design. So if you find findings from literature that explain why is it so, it would add great value to your work.

Same comment for **Line 269-271,**

**Line 281:** You suddenly talked about “Hypertensive SLT users”, but hypertension is not described in Methods and not shown in Results. Sudden claim like this are abrupt and not appropriate at the discussion section. If you think it’s key finding, please describe about it in the result section, as well as the variables section where you described each variable.

**Line 282-284:** Repetitive information. Reported on line 267. These lines can be place together if you think it’s different

**Line 296-297:** Repetitive information. Reported on line 277

**Line 300 -301:** if you would add how that part of country managed to reduce the burden, that can be one of your core recommendations for all other parts. I strongly suggest to follow my previous comments for this section.

**Line 306**: ‘influences women's use of SLT through peer effects.’ What is the basis of this claim? Your study didn’t find that. If it is from any literature, you must add reference.

Overall, the discussion section lacks in-depth exploration and explanation. This section needs to be rewritten and well referenced backings for the determinants. Please revise accordingly

**Policy Implications**

**Line 324 – 326;** Needs reference

**Line 327 – 342**: this sort of claim should be placed in recommendation section.

Policy implication and Recommendation serve different purposes in a manuscript. Policy implications explain what the findings mean for existing or potential strategies, without prescribing specific actions that is for recommendations.

Also for the recommendations, these strong throw of words must need literature support and prior empirical evidence, either within the country or in other countries. So these recommendations must be backed by references. You must add references for those, may what other countries have done to reduce the burden of SLT. If there isn’t any empirical evidence, you must adopt a **cautious and prospective tone,** use conditional language (e.g., **may*,* could*,* has the potential to**) for recommendations, clearly acknowledge the lack of empirical evidence, and position these recommendations as a **future policy research or direction grounded in theory or related evidence**.

# Verdict: Major Revision Needed

The topic is important and policy-relevant. Using three NFHS rounds to examine temporal changes and determinants of smokeless tobacco (SLT) use among women is a strength, as is the large sample and the attempt to map state variation. However, several **core methodological details are missing or unclear** (survey design handling, outcome comparability across rounds, model specification, missing data, and interpretation). The writing also needs tightening, and some claims in Discussion/Policy implications require stronger evidence or careful rephrasing.

You broadly need to work on

1) Outcome definition and cross-round comparability

2) Survey design and weighting and Analysis

4) Interpretation issues and claims

5) Presentation of results: descriptive and regression table readability, formatting as per journal guideline

6) Figure formatting as per journal guideline

6) Support of Claims in Discussion/Policy implications:

7) Separate Recommendation section

Add frequency tables and bivariate tables for each round just like you reported regression table. Revising the whole analysis would be a hassle, so I suggest you keep the regression analysis as it is. But you must revise the interpretations without cross-round comparison of aORs, write the findings individually for each round. And state “not incorporating the survey strata or PSUs in the analysis and it’s implications’ in the methodological limitation section. Please incorporate the suggestions accordingly.

I appreciate the hard works you put on this work.

Warm Regards.
