# Supplementary material for: Trends and correlates of smokeless tobacco use among Indian women: Evidence from three rounds of the National Family Health Survey 2005–2021
Source: PLoS One. 2026 Aug 3;21(8):e0353704. doi: 10.1371/journal.pone.0353704 (PMC13432099; doi:10.1371/journal.pone.0353704)
Supplement: Attachment — Submitted filename: PLOS One reviewer.docx [file pone.0353704.s006.docx]

Reviewer #3: General feedback
This is an interesting study that has its merits for publication. However, several reporting styles and information need to be updated prior for accepted.

**Comments:**
Abstract:
1. line 50: unsure why NFHS 4 not written here, bit inconsistency. If want to compare all, should put all.

**Authors reply**: Thank you this is done in the revised manuscript.
2. Would be helpful to write the year of study in the method as to contextualise the trend pattern between the three surveys

**Authors reply**: Thank you, this is done.

3. Also, unsure why only data prevalence for NFHS 3 was written.

**Authors reply**: it has been revised.
4. Line 54: Across the survey’s lower education. Older age – should not have full stop here, change it to comma

**Authors reply**: Thank you this is done.
Introduction
5. Generally, please check the placement of reference and full stop symbol. For eg ref 1 – full stop before citation. Ref 8,9 full stop after reference. Standardise this – would assume that usually full stop placed after the reference number. Worth checking

**Authors reply:** Thank you for the comment and we have now standardized this.
6. Line 74 – nations, : the sentence ends with a comma. Should change to full stop.

**Authors reply**: Thank you, this is corrected.
7. Line 79: Evidence highlight that SLT consumption in India is deeply embedded in traditional practices, with use often normalized within certain communities and social groups. – this sentence had no reference as to support the sentence. What ‘certain communities’ are normalised with. Are you referring women as to that community?

**Authors reply**: Thank you, we have provided the reference to this statement.
8. Line 101: ….. Survey (NFHS) …. – perhaps could add the word ‘in the’ before National, also ‘and’ after (NFHS)

**Authors reply**: This is done.
9. In general: good narrative was done to emphasise about the evidence of SLT prevalence and women as target group focus of research. But only rural areas and age were written as possible factors that determine SLT. Noted in method you have written the sentence in Like 126 about determinants of SLT use in India with reference 15-20. Perhaps these details need to be emphasised in the introduction as this is the main aim and written in the title (determinants). Need to say which religion, caste, education, occupation, etc had more prevalence of SLT, especially among women as the gender use in this study. With so many determinants listed here, unsure which is relevant as no evidence were detailed about the study findings. The references 15-20 need to be put in context in the introduction.

**Authors reply:** Thank you this is done.
Method
10. Line 113, perhaps the last part of this need to put references about the actual methods of the surveys. Would assume based on the narrative that this is the secondary data analyses, perhaps can emphasise that for using three national cross-sectional surveys.

**Authors reply**: This is done
11. Line 118 – worth to standardise the way numbering and comma symbol is used. Inconsistency here as shown 1,24,385 then 699,686, and 724,115

**Author reply**: Thank you for your comment, this is done.
12. Line 120: Each participant was asked about the “type of tobacco used?” who chewed tobacco in the form of khaini/gutkha/Paan masala-tobacco/Paan-tobacco those were categorized as ‘Smokeless Tobacco (SLT) user’ in all three rounds of surveys and assigned as the outcome variable of this study. - this sentence is a bit confusing. Don’t think this part is needed: who chewed tobacco in the form of.. – could replace by saying with options khaini, etc. then say that in this study. Then say that those who answered yes in any were categorised as SLT user. Assuming that you are using this variable as binary right, meaning prevalence of using any of these tobacco form vs those who did not use any SLT.

**Authors reply**: Thank you for this helpful suggestion. We agree that the description of the outcome variable was unclear and could be interpreted ambiguously. We have now revised the sentence to clearly describe the response options and the binary categorisation of smokeless tobacco use.
13. Line 158: noted that descriptive table 1 also include nutritional and behavioural factors but this sentence was saying that it only describes sociodemographic

**Authors reply**: This is done.
14. No mention of handling of missing data, or any missing data reported. Or if conducted complete case analysis by excluding any missing data. Need this information.

**Authors reply**: Thank you for this important observation. We agree that the handling of missing data was not clearly described in the manuscript. We have now added clarification in the Methods section regarding missing data management.

15. Line 159: a bit confusing about how binary logistic regression was used. Am I correct to assume that firstly, bivariate regression was conducted for outcome (yes vs no use of SLT) with each determinant? Then, those only with significant association were further adjusted in multivariable. Need better wording to say this (if I am correct) explicitly. Also, if there is any justification of why doing this sort of bivariate, then multivariate. Worth telling readers about justification.

**Authors reply**: Thank you for this valuable observation. We agree that the description of the regression modelling approach was not sufficiently explicit. We have now revised the Statistical Analysis section
16. Also noted from Supp Table 1 that some data were not available in NFHS 3 such as hypertension and smoking, but why does this was not written in the method. Now it looks as if all data were complete, all were available and comparable, and measured similarly across three survey years. Need a disclaimer if that is the case.

**Authors reply**: Thank you for this important observation. We agree that the manuscript did not sufficiently clarify differences in variable availability across the three NFHS survey rounds. We have now added a clarification in the Methods section indicating that some variables were not uniformly available. We have also added a limitation statement acknowledging that variations in variable availability and measurement across survey rounds may affect direct comparability of some findings over time.

17. Noted that multivariable adjustment will be done, but unsure how the logical justification of reference category. This could relate to absence of literature review highlighting what category used more or less SLT. For example, in religion, unsure why others were used as reference as this did not contextualise the argument of including the variable as a determinant. Which then lead to meaningful results interpretation of seeing in this paper that hindu or muslim had higher odds of using SLT than others. Quite hard to comprehend the results meanings. Same goes to region, now South is the reference, is it because the literature found that Southern has less/more SLT user. If no such studies were done before, worth mentioning that authors hypothesising that this reference category is chosen as baseline or something like that.

**Authors reply**: Reference groups were selected either based on conceptual relevance, lower prevalence of smokeless tobacco use, or to facilitate meaningful comparisons across socio-demographic groups. For example, the “Others” religion category was retained as the reference group to provide comparison across the major religious categories included in the analysis. We acknowledge that some reference category selections were methodological rather than hypothesis-driven,

18. One of the things I noticed is the word ‘reproductive age’, but no such justification of why this age criteria was used and based on what reference or guideline of cutoff age group.

**Authors reply:** Thank you, the NFHS surveys are designed to collect detailed health and demographic information among women aged 15–49 years, which is the internationally accepted demographic definition of women of reproductive age used in Demographic and Health Surveys (DHS) and related public health research. Therefore, the present study followed the standard NFHS age classification.

19. Line 156: noticed this “Microsoft Office” and “Q GIS” for the heat maps. – but upon reading the whole manuscript, could not find any results of these. Perhaps delete?

**Authors reply**: Thank you, we have now deleted this.

Results
20. Line 169: (±9.4) – is this the standard deviation, worth putting SD: in the front of the number. Also worth saying the mean age of these three surveys were almost similar, so to set the scene that sample comparison of trends was done in almost same mean age demographic

**Authors reply**: Thank you for this useful suggestion. We agree that the presentation of the mean age values was unclear. We have now explicitly indicated that the values presented alongside the mean age represent standard deviations (SD).

21. Line 170 and 171: the verb ‘are’ should be changed to ‘were’ as this is reporting the conducted study – past tense

**Authors reply**: Thank you corrected.
22. No information of combined (N) prevalence of SLT in the table as this is vital information for the main outcome of interest. Noted each type of SLT prevalence but need the number as a total use of SLT combining all types as this percentage 8.4%, 5.6%, and 3.8% did not presence in the table.

**Authors reply**: Thank you for this helpful suggestion. The overall prevalence of smokeless tobacco use among women across the three NFHS rounds was already reported in the Abstract, Results section, and illustrated in Figure 1.
23. Supp Table 1: the table heading for relative change was incorrect as what is shown is absolute change of difference by subtracting the survey 4-3 and then 5-4.

**Authors reply**: Thank you for this careful observation. We agree that the term “relative change” was inaccurately used in Supplementary Table 1. The values presented in the table represent absolute differences in prevalence between survey rounds rather than relative percentage changes. We have therefore revised the table heading to accurately reflect the analysis presented.

24. Line 173: had a bit of problem to say that observed from the figure is where you are extrapolating that it has been declined. While in absolute difference is quite small at 3% difference between NFHS 3 and 4, then 2% from NFHS 4 and 5. It is declining, which is true, but not based on the figure shown as this is scaled on y-axis at 1-9% (relatively quite small). Perhaps if you want to highlight the relative change – which is a good point as the central of trend change. Or to put disclaimer that it was a good that the trend the declining, although at a small percentage.

**Authors reply**: Thank you for this thoughtful observation. We agree that the wording may have overstated the visual interpretation of decline from the figure presentation. We have now revised the text to more cautiously describe the findings as a gradual decline in smokeless tobacco use across survey rounds.

25. Line 198: the word ‘incidence’ was not quite right as this implied that these three studies followed the same individuals over time (cohort/longitudinal study), while these are cross sectional. Perhaps change to prevalence

**Authors reply**: Thank you this is done.
26. Line 202 ; ‘are’ need to be in past tense = were.
27. Line 203 ‘is’ to was
28. Line 208 ‘is’ to was

**Authors reply:** These are corrected.

29. Line 209: State-wise prevalence of SLT use in NFHS 5. This is a good way of finding out states and prevalence of SLT for policymaker. However, this was not written as part of the aim, so having state-wise as a separate subheading was out of sudden without prior justification in the introduction. Perhaps could emphasise no studies have conducted state differences in prevalence of SLT, then this makes sense to emphasise it in the result, so it is intentional so to say to put another subheading for state analysis. Also, perhaps subheading could revise to say prevalence of SLT use according to states. Also why is only using NFHS 5, this was not mentioned in the method that you are going to do this. And by mentioning North-eastern (this is a region as you mention in method), and what are the included states in this region. The subheading was about state, but first sentence was telling about NE region. Does the figure include all states in India, or only in NE. perhaps could label figure using color coded to be more explicit what region of each of these states (assuming the figure is all states as the title of figure is general)

**Author reply**: Thank you for this detailed and constructive observation. NFHS-5 was used for this analysis because it represents the most recent nationally representative dataset. Additionally, the Results subheading has been revised to “Prevalence of smokeless tobacco use across Indian states” for improved clarity. We also revised the text to clearly distinguish between North-Eastern region findings and state-level findings.

30. Factors associated with SLT use : unsure where are the results for the unadjusted association as you mention this would be done before putting only the significant ones in the multivariable. Was this conducted or not.

**Authors reply:** Thank you for your comment this has been corrected.

31. Results from multivariable regression:
a. First sentence is repeating whats in the table about age. Would consider highlighting that from these three surveys, older adults had higher likelihood of using SLT; perhaps this is the main point.

**Authors reply**: Thank you , this is done.
b. Second point about religion, would also that Muslim higher odds of SLT use in al three surveys compared to others. Incorrect to say other religious groups as the comparison is with ‘Others’ categories, quite a distinction.

**Authors reply**: Thank you this is done.

c. Education: Women with primary education also found higher AOR in NFHS 3 (not just 5)

**Authors reply**: Corrected. Thank you.

d. Occupation: what is the similar trend, need to mention this either lower or higher among which group in comparison to those who were unemployed

**Authors reply**: Thank you for this helpful observation. We agree that the occupational trends were not sufficiently interpreted in relation to the reference category (“Not working”). We have now revised the Results.

e. BMI: no results written in the paragraph

**Authors reply:** Thank you for this important observation. We agree that the findings related to Body Mass Index (BMI) were not described in the Results section despite being presented in the regression table. We have now added interpretation of the BMI findings in the Results

32. Title Table 1 refers to only sociodemographic while the adjusted variables included nutritional and behavioural factors

**Authors reply**: Thank you for this helpful observation. We agree and have revised this.

33. Line 236: ‘are’ to ‘were’, ‘do’ change to ‘did’. Line 237: their counterparts here would mean those who were not separated and those who lived with partners. While the reference here was those who were not married. Quite a distinction.

**Authors reply**: Thank you we have now revised this section.

Discussion
34. Line 250: unsure what you want to say here about the systematic review finding of 7.7% here. Understood the way of writing discussion by including previous reported studies, but this does not explain anything or contextualise the number. Could say if it was different method of included studies in the systematic review, or year of studies included, does those studies only conducted among women, etc. Also did not understand why do you say…some previous Indian studies reported higher prevalence of SLT. Was it necessary, also what is the prevalence, was it using the same dataset, measured similarly, with what included types of SLT in the studies

**Authors reply**: Thank you, we have now clarified and made the suggested edits.

35. Line 252: The use of Guthka….. again here understood the key finding, but unsure how it relates to cite the consistent with prior study. So then what, does it validate your findings, or does it highlight the issue of SLT, is it different because of what, did not understand the point you’re making here besides quoting previous studies.

**Authors reply**: Thank you, this is revised.
36. Line 259. Perhaps need a full stop somewhere here because sentence structure is quite confusing.

**Authors reply**: Thank you have edited.
37. Line 266: indicates the willingness to quit….unsure where is the evidence, or is it studies from 28,29 showed that willingness to quit was higher among rural area women. The sentence structure needs to be improved

**Authors reply**: Thank you for this important observation. We have therefore revised the sentence.

38. Line 271, need a full stop symbol after the word survey.

**Authors reply**: This is done.
39. Line 272-Line 277: all are written without in depth analytical explanation of why such trends, instead of just repeating what was found. ..attributed to cultural influences had no evidence placed here

**Authors reply**: Thank you , we have now revised this section with references.

40. Line 280: This might be attributed to a lack of knowledge and different perspectives on SLT use in community.. unsupported claims. Sounds like authors are hypothesising it in one sentence of why such patterns.

**Authors reply**: Thank you for this important observation. We have therefore revised the sentence to avoid unsupported causal interpretation and to present the explanation more cautiously and contextually.

41. Line 308: burden. . two full stops were written here

**Authors reply**: This is done.

Reviewer #4: I was a pleasure to review this manuscript which addresses a community issue that has not been adequately explored previously.
I have few comments:

Page7, line 189-190 : whereas there is a sharp rise… etc. It is a bit unclear what do you mean by this sentence is it a rise in SLT prev in survey 4 compared to 3 or compared to 5? I suggest you rewrite this sentence.

**Authors reply**: This is done, we thank the reviewer.

The outcome is not clear. You need to clarify whether the frequency and period of consumption was recorded? As you may know that infrequent usage of tobacco does not have the same effect as frequent you. In smoking, we use ( pack per year) to understand this relationship. I’m not sure whether there is any similar method for SLT.

**Author reply:** Thank you for this important observation. We agree that the description of the outcome variable required further clarification regarding the measurement of smokeless tobacco use. The NFHS datasets used in this study captured current use of specific smokeless tobacco products but did not provide detailed information on frequency, duration, or cumulative exposure of SLT consumption comparable to measures such as “pack-years” used in smoking research. Therefore, the outcome variable in the present study was defined as a binary measure indicating whether women reported current use of any smokeless tobacco product.

Another comment raised to my concern, are those 3 surveys uses the same population? Maybe the decrease is due to deceased people. Was there a difference in the prevalence between age groups among the three surveys? For example in survey one young age and in the following surveys were older?

**Authors reply**: Thank you for this important methodological observation. We would like to clarify that NFHS-3, NFHS-4, and NFHS-5 are independent repeated cross-sectional surveys and do not follow the same individuals over time. Therefore, the observed decline in smokeless tobacco use does not reflect changes due to death or follow-up of the same population, but rather represents population-level prevalence estimates from nationally representative samples collected during different survey periods.

We agree that differences in age distribution across surveys could potentially influence prevalence estimates. However, the mean age distribution of women was broadly similar across the three NFHS rounds, supporting comparability across survey years. Additionally, age-stratified analyses and multivariable regression models adjusting for age were conducted to account for differences in demographic composition between survey rounds.

Thank you
