# Supplementary material for: Trends and correlates of smokeless tobacco use among Indian women: Evidence from three rounds of the National Family Health Survey 2005–2021
Source: PLoS One. 2026 Aug 3;21(8):e0353704. doi: 10.1371/journal.pone.0353704 (PMC13432099; doi:10.1371/journal.pone.0353704)
Supplement: Peer review history

# Peer review: Decision Letter 0

**DOI:** 10.1371/journal.pone.0353704.r001

**Authors:** Bijit Biswas

28 Jan 2026

PONE-D-25-25520

Trends and determinants of smokeless tobacco use among Indian women: Evidence from three rounds of the National Family Health Survey 2005-2021

PLOS One

Dear Dr. Goel,

Thank you for submitting your manuscript to PLOS ONE. After careful consideration, we feel that it has merit but does not fully meet PLOS ONE’s publication criteria as it currently stands. Therefore, we invite you to submit a revised version of the manuscript that addresses the points raised during the review process.

==============================

**ACADEMIC EDITOR:**  This is an important and timely manuscript on smokeless tobacco use among Indian women, and it is generally well written with appropriate analysis. However, minor revisions are needed: strengthen the rationale for focusing on women of reproductive age, clarify handling of missing data and potential multicollinearity in the models, consider reporting model diagnostics, and provide 95% confidence intervals for prevalence estimates. Please also reconcile inconsistencies between descriptive and regression findings (e.g., rural–urban differences), expand discussion around key trends (such as rising urban use in NFHS-5), rephrase overly broad claims, and carefully revise language and formatting throughout.

==============================

Please submit your revised manuscript by Mar 14 2026 11:59PM. If you will need more time than this to complete your revisions, please reply to this message or contact the journal office at plosone@plos.org. When you're ready to submit your revision, log on to https://www.editorialmanager.com/pone/ and select the 'Submissions Needing Revision' folder to locate your manuscript file.

Please include the following items when submitting your revised manuscript:

- A letter that responds to each point raised by the academic editor and reviewer(s). You should upload this letter as a separate file labeled 'Response to Reviewers'.
- A marked-up copy of your manuscript that highlights changes made to the original version. You should upload this as a separate file labeled 'Revised Manuscript with Track Changes'.
- An unmarked version of your revised paper without tracked changes. You should upload this as a separate file labeled 'Manuscript'.

-->If you would like to make changes to your financial disclosure, please include your updated statement in your cover letter. Guidelines for resubmitting your figure files are available below the reviewer comments at the end of this letter.

If applicable, we recommend that you deposit your laboratory protocols in protocols.io to enhance the reproducibility of your results. Protocols.io assigns your protocol its own identifier (DOI) so that it can be cited independently in the future. For instructions see: https://journals.plos.org/plosone/s/submission-guidelines#loc-laboratory-protocols. Additionally, PLOS ONE offers an option for publishing peer-reviewed Lab Protocol articles, which describe protocols hosted on protocols.io. Read more information on sharing protocols at https://plos.org/protocols?utm_medium=editorial-email&utm_source=authorletters&utm_campaign=protocols.

We look forward to receiving your revised manuscript.

Kind regards,

Bijit Biswas, MBBS, MD, DNB

Academic Editor

PLOS One

Journal Requirements:

When submitting your revision, we need you to address these additional requirements.

1. Please ensure that your manuscript meets PLOS ONE's style requirements, including those for file naming. The PLOS ONE style templates can be found at

https://journals.plos.org/plosone/s/file?id=wjVg/PLOSOne_formatting_sample_main_body.pdf and

https://journals.plos.org/plosone/s/file?id=ba62/PLOSOne_formatting_sample_title_authors_affiliations.pdf

2. We noticed you have some minor occurrence of overlapping text with the following previous publication(s), which needs to be addressed:

- -doi: 10.1186/s12916-022-02662-0

In your revision ensure you cite all your sources (including your own works), and quote or rephrase any duplicated text outside the methods section. Further consideration is dependent on these concerns being addressed.

3. Thank you for stating the following in the Competing Interests/Financial Disclosure section:

The authors have declared that no competing interests exist.

We note that one or more of the authors are employed by a commercial company: VART Consulting Pvt. Ltd

a. Please provide an amended Funding Statement declaring this commercial affiliation, as well as a statement regarding the Role of Funders in your study. If the funding organization did not play a role in the study design, data collection and analysis, decision to publish, or preparation of the manuscript and only provided financial support in the form of authors' salaries and/or research materials, please review your statements relating to the author contributions, and ensure you have specifically and accurately indicated the role(s) that these authors had in your study. You can update author roles in the Author Contributions section of the online submission form.

Please also include the following statement within your amended Funding Statement.

“The funder provided support in the form of salaries for authors [insert relevant initials], but did not have any additional role in the study design, data collection and analysis, decision to publish, or preparation of the manuscript. The specific roles of these authors are articulated in the ‘author contributions’ section.”

If your commercial affiliation did play a role in your study, please state and explain this role within your updated Funding Statement.

b. Please also provide an updated Competing Interests Statement declaring this commercial affiliation along with any other relevant declarations relating to employment, consultancy, patents, products in development, or marketed products, etc.

Within your Competing Interests Statement, please confirm that this commercial affiliation does not alter your adherence to all PLOS ONE policies on sharing data and materials by including the following statement: "This does not alter our adherence to PLOS ONE policies on sharing data and materials.” (as detailed online in our guide for authors http://journals.plos.org/plosone/s/competing-interests) . If this adherence statement is not accurate and there are restrictions on sharing of data and/or materials, please state these. Please note that we cannot proceed with consideration of your article until this information has been declared.

Please include both an updated Funding Statement and Competing Interests Statement in your cover letter. We will change the online submission form on your behalf.

4. We note that Figure 2 in your submission contain map images which may be copyrighted. All PLOS content is published under the Creative Commons Attribution License (CC BY 4.0), which means that the manuscript, images, and Supporting Information files will be freely available online, and any third party is permitted to access, download, copy, distribute, and use these materials in any way, even commercially, with proper attribution. For these reasons, we cannot publish previously copyrighted maps or satellite images created using proprietary data, such as Google software (Google Maps, Street View, and Earth). For more information, see our copyright guidelines: http://journals.plos.org/plosone/s/licenses-and-copyright.

We require you to either (a) present written permission from the copyright holder to publish these figures specifically under the CC BY 4.0 license, or (b) remove the figures from your submission:

a. You may seek permission from the original copyright holder of Figure 2 to publish the content specifically under the CC BY 4.0 license.

We recommend that you contact the original copyright holder with the Content Permission Form (http://journals.plos.org/plosone/s/file?id=7c09/content-permission-form.pdf) and the following text:

“I request permission for the open-access journal PLOS ONE to publish XXX under the Creative Commons Attribution License (CCAL) CC BY 4.0 (http://creativecommons.org/licenses/by/4.0/). Please be aware that this license allows unrestricted use and distribution, even commercially, by third parties. Please reply and provide explicit written permission to publish XXX under a CC BY license and complete the attached form.”

Please upload the completed Content Permission Form or other proof of granted permissions as an ""Other"" file with your submission.

In the figure caption of the copyrighted figure, please include the following text: “Reprinted from [ref] under a CC BY license, with permission from [name of publisher], original copyright [original copyright year].”

b. If you are unable to obtain permission from the original copyright holder to publish these figures under the CC BY 4.0 license or if the copyright holder’s requirements are incompatible with the CC BY 4.0 license, please either i) remove the figure or ii) supply a replacement figure that complies with the CC BY 4.0 license. Please check copyright information on all replacement figures and update the figure caption with source information. If applicable, please specify in the figure caption text when a figure is similar but not identical to the original image and is therefore for illustrative purposes only.

The following resources for replacing copyrighted map figures may be helpful:

USGS National Map Viewer (public domain): http://viewer.nationalmap.gov/viewer/

The Gateway to Astronaut Photography of Earth (public domain): http://eol.jsc.nasa.gov/sseop/clickmap/

Maps at the CIA (public domain): https://www.cia.gov/library/publications/the-world-factbook/index.html and https://www.cia.gov/library/publications/cia-maps-publications/index.html

NASA Earth Observatory (public domain): http://earthobservatory.nasa.gov/

Landsat: http://landsat.visibleearth.nasa.gov/

USGS EROS (Earth Resources Observatory and Science (EROS) Center) (public domain): http://eros.usgs.gov/#

Natural Earth (public domain): http://www.naturalearthdata.com/

5. Please include captions for your Supporting Information files at the end of your manuscript, and update any in-text citations to match accordingly. Please see our Supporting Information guidelines for more information: http://journals.plos.org/plosone/s/supporting-information.

6. If the reviewer comments include a recommendation to cite specific previously published works, please review and evaluate these publications to determine whether they are relevant and should be cited. There is no requirement to cite these works unless the editor has indicated otherwise.

7. Please review your reference list to ensure that it is complete and correct. If you have cited papers that have been retracted, please include the rationale for doing so in the manuscript text, or remove these references and replace them with relevant current references. Any changes to the reference list should be mentioned in the rebuttal letter that accompanies your revised manuscript. If you need to cite a retracted article, indicate the article’s retracted status in the References list and also include a citation and full reference for the retraction notice.

Additional Editor Comments:

This is an important and timely manuscript on smokeless tobacco use among Indian women, and it is generally well written with appropriate analysis. However, minor revisions are needed: strengthen the rationale for focusing on women of reproductive age, clarify handling of missing data and potential multicollinearity in the models, consider reporting model diagnostics, and provide 95% confidence intervals for prevalence estimates. Please also reconcile inconsistencies between descriptive and regression findings (e.g., rural–urban differences), expand discussion around key trends (such as rising urban use in NFHS-5), rephrase overly broad claims, and carefully revise language and formatting throughout.

[Note: HTML markup is below. Please do not edit.]

Reviewers' comments:

Reviewer's Responses to Questions

**Comments to the Author**

1. Is the manuscript technically sound, and do the data support the conclusions?

The manuscript must describe a technically sound piece of scientific research with data that supports the conclusions. Experiments must have been conducted rigorously, with appropriate controls, replication, and sample sizes. The conclusions must be drawn appropriately based on the data presented.

Reviewer #1: Yes

Reviewer #2: Partly

**********

2. Has the statistical analysis been performed appropriately and rigorously?

Reviewer #1: Yes

Reviewer #2: No

**********

3. Have the authors made all data underlying the findings in their manuscript fully available?

The PLOS Data policy requires authors to make all data underlying the findings described in their manuscript fully available without restriction, with rare exception (please refer to the Data Availability Statement in the manuscript PDF file). The data should be provided as part of the manuscript or its supporting information, or deposited to a public repository. For example, in addition to summary statistics, the data points behind means, medians and variance measures should be available. If there are restrictions on publicly sharing data—e.g. participant privacy or use of data from a third party—those must be specified.

Reviewer #1: Yes

Reviewer #2: Yes

**********

4. Is the manuscript presented in an intelligible fashion and written in standard English?

PLOS ONE does not copyedit accepted manuscripts, so the language in submitted articles must be clear, correct, and unambiguous. Any typographical or grammatical errors should be corrected at revision, so please note any specific errors here.

Reviewer #1: Yes

Reviewer #2: No

**********

5. Review Comments to the Author

Please use the space provided to explain your answers to the questions above. You may also include additional comments for the author, including concerns about dual publication, research ethics, or publication ethics. (Please upload your review as an attachment if it exceeds 20,000 characters)

Reviewer #1: Dear Authors, thank you for a very interesting and important manuscript on smokeless tobacco use among women aged 15-49 years in India. The manuscript is generally well written and adds a valuable contribution to the ongoing tobacco control debates in India. I have a few comments that I believe will help enhance your work. I have provided them section by section for ease of reference.

Introduction

The introduction while provides a broader picture, I have the following observations which the authors may consider to address

i) It does not satisfactorily narrow down on why it’s important to look into the SLT use among women of reproductive age.

ii) Discussing on the existing gaps in tobacco control efforts and how women who use SLT, particularly those belonging to this age group are missed out might help to build the context.

iii) The specific independent variables and their inclusion is not adequately rationalized in the introduction section. As a suggestion it may be good to provide a conceptual framework given that the study is reliant on secondary analysis. Such a framework could help to justify the choice of selected factors associated.

Methodology

i) It was unclear how the researchers addressed missing data. Providing information about the same would be useful.

ii) There is a potential that several of independent variables could have multicollinearity and effect the results of multivariable models, did the authors test for it? Providing the details of the same would be valuable.

iii) Were any post-test estimates for regression models were computed (for example goodness of fit tests, sensitivity and specificity of models, and AUROC estimates)? If yes please provide them. If they were not done it might be good to check as large survey data have the issue of identifying highly significant associations but weak models.

iv) I assume that the authors have not pooled the samples from NFHS-3 to NFHS-5, and have descriptively reported the trend. While I am not advicing on pooling, I am curious to know if the authors have considered to do so as it has its advantages (eg. Assess the statistical significance of the changing trend in SLT prevalence).

Results

i) Please provide 95% CI for the prevalence estimates in the text.

ii) The results from supplementary table does not seem to sync well with the results of multivariate table in some areas. With regard to the place of residence, rural regions had higher proportion of women using SLT in NFHS 3 & 4, whereas the odds seem to show that rural women had a lower probability of using SLT.

Discussion

i) Each of the findings could be better discussed. For example with reference to association between place of residence and SLT use. Its good that the researchers reported that the urban areas had higher SLT use during NFHS-5, but what could have led to this raise? Was it migration? COVID-19? Targeted SLT control interventions in states where it is highly prevalent ? discussion around this direction strengthen the arguments.

ii) The statements like ‘however the eastern part of country somehow managed to reduce the burden of SLT in last three rounds’ seems to be very loose. Rather discussing on what strategies could have contributed to this would be very helpful and might show a means for expansion to the regions where SLT persists to be high.

Methodological limitation

i) “to our knowledge, this is the first study that contributes to understanding the trends of SLT usage among women using the latest NFHS surveys”. I have come across the studies that have done this using latest NFHS rounds. It might be good to remove/reword this statement as it may not add much.

ii) To the limitation it may be better to add the context of social desirability bias, specifically given that the NFHS-5 has been conducted during the COVID-19 years, chewing tobacco use could have been looked on from a stigmatized lens so people may not have reported even if they consumed.

General comment

The writing can be improved. Please carefully check the grammar, formatting, font differences and typographic errors in the manuscript.

Reviewer #2: The topic is important and policy-relevant. Using three NFHS rounds to examine temporal changes and determinants of smokeless tobacco (SLT) use among women is a strength, as is the large sample and the attempt to map state variation. However, several core methodological details are missing or unclear (survey design handling, outcome comparability across rounds, model specification, missing data, and interpretation). The writing also needs tightening, and some claims in Discussion/Policy implications require stronger evidence or careful rephrasing.

You broadly need to work on

1) Outcome definition and cross-round comparability

2) Survey design and weighting and Analysis

4) Interpretation issues and claims

5) Presentation of results: descriptive and regression table readability, formatting as per journal guideline

6) Figure formatting as per journal guideline

6) Support of Claims in Discussion/Policy implications:

7) Separate Recommendation section

Add frequency tables and bivariate tables for each round just like you reported regression table. Revising the whole analysis would be a hassle, so I suggest you keep the regression analysis as it is. But you must revise the interpretations without cross-round comparison of aORs, write the findings individually for each round. And state “not incorporating the survey strata or PSUs in the analysis and it’s implications’ in the methodological limitation section. Please incorporate the suggestions accordingly. See details in the attached document.

I appreciate the hard works you put on this work.

Warm Regards.

**********

6. PLOS authors have the option to publish the peer review history of their article (what does this mean?). If published, this will include your full peer review and any attached files.

If you choose “no”, your identity will remain anonymous but your review may still be made public.

**Do you want your identity to be public for this peer review?**  For information about this choice, including consent withdrawal, please see our Privacy Policy.

Reviewer #1: No

Reviewer #2: **Yes:**  Dr. Tanvir Ahmed

**********

[NOTE: If reviewer comments were submitted as an attachment file, they will be attached to this email and accessible via the submission site. Please log into your account, locate the manuscript record, and check for the action link "View Attachments". If this link does not appear, there are no attachment files.]

To ensure your figures meet our technical requirements, please review our figure guidelines: https://journals.plos.org/plosone/s/figures

You may also use PLOS’s free figure tool, NAAS, to help you prepare publication quality figures: https://journals.plos.org/plosone/s/figures#loc-tools-for-figure-preparation.

NAAS will assess whether your figures meet our technical requirements by comparing each figure against our figure specifications.

Attachment Submitted filename: Review Comments for PLOS SLT article.docx

Attachment Submitted filename: Review on PONE-D-25-25520.docx

# Author comment: Author response to Decision Letter 1

**DOI:** 10.1371/journal.pone.0353704.r002

10 Mar 2026

Dear Academic Editor/Reviewers,

We, thank the reviewers and editors for the detailed review. Kindly find the rebuttal letter attached with revised with response to each point raised by your good self.

Regards

Attachment Submitted filename: Author reply.docx

# Peer review: Decision Letter 1

**DOI:** 10.1371/journal.pone.0353704.r003

**Authors:** Bijit Biswas

24 May 2026

PONE-D-25-25520R1

Trends and determinants of smokeless tobacco use among Indian women: Evidence from three rounds of the National Family Health Survey 2005-2021

PLOS One

Dear Dr. Goel,

Thank you for submitting your manuscript to PLOS ONE. After careful consideration, we feel that it has merit but does not fully meet PLOS ONE’s publication criteria as it currently stands. Therefore, we invite you to submit a revised version of the manuscript that addresses the points raised during the review process.

==============================

**ACADEMIC EDITOR:**  The revised manuscript addresses an important public health issue and shows substantial improvement. However, several concerns related to reporting consistency, methodological clarity, justification of analytical approaches, handling of missing data, and interpretation of findings still require minor revision before acceptance. Greater clarity in the Introduction, Methods, and Discussion sections would further strengthen the manuscript.

==============================

Please submit your revised manuscript by Jul 08 2026 11:59PM. If you will need more time than this to complete your revisions, please reply to this message or contact the journal office at plosone@plos.org. When you're ready to submit your revision, log on to https://www.editorialmanager.com/pone/ and select the 'Submissions Needing Revision' folder to locate your manuscript file.

Please include the following items when submitting your revised manuscript:

- A letter that responds to each point raised by the academic editor and reviewer(s). You should upload this letter as a separate file labeled 'Response to Reviewers'.
- A marked-up copy of your manuscript that highlights changes made to the original version. You should upload this as a separate file labeled 'Revised Manuscript with Track Changes'.
- An unmarked version of your revised paper without tracked changes. You should upload this as a separate file labeled 'Manuscript'.

If you would like to make changes to your financial disclosure, please include your updated statement in your cover letter. Guidelines for resubmitting your figure files are available below the reviewer comments at the end of this letter.

If applicable, we recommend that you deposit your laboratory protocols in protocols.io to enhance the reproducibility of your results. Protocols.io assigns your protocol its own identifier (DOI) so that it can be cited independently in the future. For instructions see: https://journals.plos.org/plosone/s/submission-guidelines#loc-laboratory-protocols. Additionally, PLOS ONE offers an option for publishing peer-reviewed Lab Protocol articles, which describe protocols hosted on protocols.io. Read more information on sharing protocols at https://plos.org/protocols?utm_medium=editorial-email&utm_source=authorletters&utm_campaign=protocols.

As the corresponding author, your ORCID iD is verified in the submission system and will appear in the published article. PLOS supports the use of ORCID, and we encourage all coauthors to register for an ORCID iD and use it as well. Please encourage your coauthors to verify their ORCID iD within the submission system before final acceptance, as unverified ORCID iDs will not appear in the published article. *Only*  the individual author can complete the verification step; PLOS staff *cannot*  verify ORCID iDs on behalf of authors.

We look forward to receiving your revised manuscript.

Kind regards,

Bijit Biswas, MBBS, MD, DNB

Academic Editor

PLOS One

**Journal Requirements:**

1. If the reviewer comments include a recommendation to cite specific previously published works, please review and evaluate these publications to determine whether they are relevant and should be cited. There is no requirement to cite these works unless the editor has indicated otherwise.

2. Please review your reference list to ensure that it is complete and correct. If you have cited papers that have been retracted, please include the rationale for doing so in the manuscript text, or remove these references and replace them with relevant current references. Any changes to the reference list should be mentioned in the rebuttal letter that accompanies your revised manuscript. If you need to cite a retracted article, indicate the article’s retracted status in the References list and also include a citation and full reference for the retraction notice.

**Additional Editor Comments:**

The revised manuscript addresses an important public health issue and shows substantial improvement. However, several concerns related to reporting consistency, methodological clarity, justification of analytical approaches, handling of missing data, and interpretation of findings still require minor revision before acceptance.

[Note: HTML markup is below. Please do not edit.]

Reviewers' comments:

Reviewer's Responses to Questions

**Comments to the Author**

1. If the authors have adequately addressed your comments raised in a previous round of review and you feel that this manuscript is now acceptable for publication, you may indicate that here to bypass the “Comments to the Author” section, enter your conflict of interest statement in the “Confidential to Editor” section, and submit your "Accept" recommendation.

Reviewer #1: All comments have been addressed

Reviewer #3: (No Response)

Reviewer #4: (No Response)

**********

2. Is the manuscript technically sound, and do the data support the conclusions?

The manuscript must describe a technically sound piece of scientific research with data that supports the conclusions. Experiments must have been conducted rigorously, with appropriate controls, replication, and sample sizes. The conclusions must be drawn appropriately based on the data presented.

Reviewer #1: Yes

Reviewer #3: Partly

Reviewer #4: Yes

**********

3. Has the statistical analysis been performed appropriately and rigorously?

Reviewer #1: Yes

Reviewer #3: Yes

Reviewer #4: Yes

**********

4. Have the authors made all data underlying the findings in their manuscript fully available?

The PLOS Data policy requires authors to make all data underlying the findings described in their manuscript fully available without restriction, with rare exception (please refer to the Data Availability Statement in the manuscript PDF file). The data should be provided as part of the manuscript or its supporting information, or deposited to a public repository. For example, in addition to summary statistics, the data points behind means, medians and variance measures should be available. If there are restrictions on publicly sharing data—e.g. participant privacy or use of data from a third party—those must be specified.

Reviewer #1: No

Reviewer #3: Yes

Reviewer #4: (No Response)

**********

5. Is the manuscript presented in an intelligible fashion and written in standard English?

PLOS ONE does not copyedit accepted manuscripts, so the language in submitted articles must be clear, correct, and unambiguous. Any typographical or grammatical errors should be corrected at revision, so please note any specific errors here.

Reviewer #1: Yes

Reviewer #3: Yes

Reviewer #4: Yes

**********

6. Review Comments to the Author

Please use the space provided to explain your answers to the questions above. You may also include additional comments for the author, including concerns about dual publication, research ethics, or publication ethics. (Please upload your review as an attachment if it exceeds 20,000 characters)

Reviewer #1: Dear Authors, thank you all my comments have been satisfactorily addressed. While I do not have any additional comments, I would advice to kindly check for the adherence to PLOS ONE data sharing guidelines.

Reviewer #3: General feedback

This is an interesting study that has its merits for publication. However, several reporting styles and information need to be updated prior for accepted.

Comments:

Abstract:

1. line 50: unsure why NFHS 4 not written here, bit inconsistency. If want to compare all, should put all.

2. Would be helpful to write the year of study in the method as to contextualise the trend pattern between the three surveys

3. Also, unsure why only data prevalence for NFHS 3 was written.

4. Line 54: Across the survey’s lower education. Older age – should not have full stop here, change it to comma

Introduction

5. Generally, please check the placement of reference and full stop symbol. For eg ref 1 – full stop before citation. Ref 8,9 full stop after reference. Standardise this – would assume that usually full stop placed after the reference number. Worth checking

6. Line 74 – nations, : the sentence ends with a comma. Should change to full stop.

7. Line 79: Evidence highlight that SLT consumption in India is deeply embedded in traditional practices, with use often normalized within certain communities and social groups. – this sentence had no reference as to support the sentence. What ‘certain communities’ are normalised with. Are you referring women as to that community.

8. Line 101: ….. Survey (NFHS) …. – perhaps could add the word ‘in the’ before National, also ‘and’ after (NFHS)

9. In general: good narrative was done to emphasise about the evidence of SLT prevalence and women as target group focus of research. But only rural areas and age were written as possible factors that determine SLT. Noted in method you have written the sentence in Like 126 about determinants of SLT use in India with reference 15-20. Perhaps these details need to be emphasised in the introduction as this is the main aim and written in the title (determinants). Need to say which religion, caste, education, occupation, etc had more prevalence of SLT, especially among women as the gender use in this study. With so many determinants listed here, unsure which is relevant as no evidence were detailed about the study findings. The references 15-20 need to be put in context in the introduction.

Method

10. Line 113, perhaps the last part of this need to put references about the actual methods of the surveys. Would assume based on the narrative that this is the secondary data analyses, perhaps can emphasise that for using three national cross-sectional surveys.

11. Line 118 – worth to standardise the way numbering and comma symbol is used. Inconsistency here as shown 1,24,385 then 699,686, and 724,115

12. Line 120: Each participant was asked about the “type of tobacco used?” who chewed tobacco in the form of khaini/gutkha/Paan masala-tobacco/Paan-tobacco those were categorized as ‘Smokeless Tobacco (SLT) user’ in all three rounds of surveys and assigned as the outcome variable of this study. - this sentence is a bit confusing. Don’t think this part is needed: who chewed tobacco in the form of.. – could replace by saying with options khaini, etc. then say that in this study. Then say that those who answered yes in any were categorised as SLT user. Assuming that you are using this variable as binary right, meaning prevalence of using any of these tobacco form vs those who did not use any SLT.

13. Line 158: noted that descriptive table 1 also include nutritional and behavioural factors but this sentence was saying that it only describes sociodemographic

14. No mention of handling of missing data, or any missing data reported. Or if conducted complete case analysis by excluding any missing data. Need this information.

15. Line 159: a bit confusing about how binary logistic regression was used. Am I correct to assume that firstly, bivariate regression was conducted for outcome (yes vs no use of SLT) with each determinant. Then, those only with significant association were further adjusted in multivariable. Need better wording to say this (if I am correct) explicitly. Also, if there is any justification of why doing this sort of bivariate, then multivariate. Worth telling readers about justification.

16. Also noted from Supp Table 1 that some data were not available in NFHS 3 such as hypertension and smoking, but why does this was not written in the method. Now it looks as if all data were complete, all were available and comparable, and measured similarly across three survey years. Need a disclaimer if that is the case.

17. Noted that multivariable adjustment will be done, but unsure how the logical justification of reference category. This could relate to absence of literature review highlighting what category used more or less SLT. For example in religion, unsure why others were used as reference as this did not contextualise the argument of including the variable as a determinant. Which then lead to meaningful results interpretation of seeing in this paper that hindu or muslim had higher odds of using SLT than others. Quite hard to comprehend the results meanings. Same goes to region, now South is the reference, is it because the literature found that Southern has less/more SLT user. If no such studies were done before, worth mentioning that authors hypothesising that this reference category is chosen as baseline or something like that.

18. One of the things I noticed is the word ‘reproductive age’, but no such justification of why this age criteria was used and based on what reference or guideline of cutoff age group.

19. Line 156: noticed this “Microsoft Office” and “Q GIS” for the heat maps. – but upon reading the whole manuscript, could not find any results of these. Perhaps delete?

Results

20. Line 169: (±9.4) – is this the standard deviation, worth putting SD: in the front of the number. Also worth saying the mean age of these three surveys were almost similar, so to set the scene that sample comparison of trends was done in almost same mean age demographic

21. Line 170 and 171: the verb ‘are’ should be changed to ‘were’ as this is reporting the conducted study – past tense

22. No information of combined (N) prevalence of SLT in the table as this is vital information for the main outcome of interest. Noted each type of SLT prevalence but need the number as a total use of SLT combining all types as this percentage 8.4%, 5.6%, and 3.8% did not presence in the table.

23. Supp Table 1: the table heading for relative change was incorrect as what is shown is absolute change of difference by subtracting the survey 4-3 and then 5-4.

24. Line 173: had a bit of problem to say that observed from the figure is where you are extrapolating that it has been declined. While in absolute difference is quite small at 3% difference between NFHS 3 and 4, then 2% from NFHS 4 and 5. It is declining, which is true, but not based on the figure shown as this is scaled on y-axis at 1-9% (relatively quite small). Perhaps if you want to highlight the relative change – which is a good point as the central of trend change. Or to put disclaimer that it was a good that the trend the declining, although at a small percentage.

25. Line 198: the word ‘incidence’ was not quite right as this implied that these three studies followed the same individuals over time (cohort/longitudinal study), while these are cross sectional. Perhaps change to prevalence

26. Line 202 ; ‘are’ need to be in past tense = were.

27. Line 203 ‘is’ to was

28. Line 208 ‘is’ to was

29. Line 209: State-wise prevalence of SLT use in NFHS 5. This is a good way of finding out states and prevalence of SLT for policymaker. However, this was not written as part of the aim, so having state-wise as a separate subheading was out of sudden without prior justification in the introduction. Perhaps could emphasise no studies have conducted state differences in prevalence of SLT, then this makes sense to emphasise it in the result, so it is intentional so to say to put another subheading for state analysis. Also, perhaps subheading could revise to say prevalence of SLT use according to states. Also why is only using NFHS 5, this was not mentioned in the method that you are going to do this. And by mentioning North-eastern (this is a region as you mention in method), and what are the included states in this region. The subheading was about state, but first sentence was telling about NE region. Does the figure include all states in India, or only in NE. perhaps could label figure using color coded to be more explicit what region of each of these states (assuming the figure is all states as the title of figure is general)

30. Factors associated with SLT use : unsure where are the results for the unadjusted association as you mention this would be done before putting only the significant ones in the multivariable. Was this conducted or not.

31. Results from multivariable regression:

a. First sentence is repeating whats in the table about age. Would consider highlighting that from these three surveys, older adults had higher likelihood of using SLT; perhaps this is the main point.

b. Second point about religion, would also that Muslim higher odds of SLT use in al three surveys compared to others. Incorrect to say other religious groups as the comparison is with ‘Others’ categories, quite a distinction.

c. Education: Women with primary education also found higher AOR in NFHS 3 (not just 5)

d. Occupation: what is the similar trend, need to mention this either lower or higher among which group in comparison to those who were unemployed

e. BMI: no results written in the paragraph

32. Title Table 1 refers to only sociodemographic while the adjusted variables included nutritional and behavioural factors

33. Line 236: ‘are’ to ‘were’, ‘do’ change to ‘did’. Line 237: their counterparts here would mean those who were not separated and those who lived with partners. While the reference here was those who were not married. Quite a distinction.

Discussion

34. Line 250: unsure what you want to say here about the systematic review finding of 7.7% here. Understood the way of writing discussion by including previous reported studies, but this does not explain anything or contextualise the number. Could say if it was different method of included studies in the systematic review, or year of studies included, does those studies only conducted among women, etc. Also did not understand why do you say…some previous Indian studies reported higher prevalence of SLT. Was it necessary, also what is the prevalence, was it using the same dataset, measured similarly, with what included types of SLT in the studies

35. Line 252: The use of Guthka….. again here understood the key finding, but unsure how it relates to cite the consistent with prior study. So then what, does it validate your findings, or does it highlight the issue of SLT, is it different because of what, did not understand the point you’re making here besides quoting previous studies.

36. Line 259. Perhaps need a full stop somewhere here because sentence structure is quite confusing.

37. Line 266: indicates the willingness to quit….unsure where is the evidence, or is it studies from 28,29 showed that willingness to quit was higher among rural area women. The sentence structure needs to be improved

38. Line 271, need a full stop symbol after the word survey.

39. Line 272-Line 277: all are written without in depth analytical explanation of why such trends, instead of just repeating what was found. ..attributed to cultural influences had no evidence placed here

40. Line 280: This might be attributed to a lack of knowledge and different perspectives on SLT use in community.. unsupported claims. Sounds like authors are hypothesising it in one sentence of why such patterns.

41. Line 308: burden. . two full stops were written here

Reviewer #4: I was a pleasure to review this manuscript which addresses a community issue that has not been adequately explored previously.

I have few comments:

Page7, line 189-190 : whereas there is a sharp rise… etc. It is a bit unclear what do you mean by this sentence is it a rise in SLT prev in survey 4 compared to 3 or compared to 5? I suggest you rewrite this sentence.

The outcome is not clear. You need to clarify whether the frequency and period of consumption was recorded? As you may know that infrequent usage of tobacco does not have the same effect as frequent you. In smoking, we use ( pack per year) to understand this relationship. I’m not sure whether there is any similar method for SLT.

Another comment raised to my concern, are those 3 surveys uses the same population? Maybe the decrease is due to deceased people. Was there a difference in the prevalence between age groups among the three surveys? For example in survey one young age and in the following surveys were older?

Thank you

**********

7. PLOS authors have the option to publish the peer review history of their article (what does this mean?). If published, this will include your full peer review and any attached files.

If you choose “no”, your identity will remain anonymous but your review may still be made public.

**Do you want your identity to be public for this peer review?**  For information about this choice, including consent withdrawal, please see our Privacy Policy.

Reviewer #1: No

Reviewer #3: No

Reviewer #4: No

**********

[NOTE: If reviewer comments were submitted as an attachment file, they will be attached to this email and accessible via the submission site. Please log into your account, locate the manuscript record, and check for the action link "View Attachments". If this link does not appear, there are no attachment files.]

To ensure your figures meet our technical requirements, please review our figure guidelines: https://journals.plos.org/plosone/s/figures

You may also use PLOS’s free figure tool, NAAS, to help you prepare publication quality figures: https://journals.plos.org/plosone/s/figures#loc-tools-for-figure-preparation.

NAAS will assess whether your figures meet our technical requirements by comparing each figure against our figure specifications.

# Author comment: Author response to Decision Letter 2

**DOI:** 10.1371/journal.pone.0353704.r004

29 May 2026

We thank the Editor and Reviewers for their valuable comments and suggestions, which helped improve the quality and clarity of the manuscript. We have carefully addressed all comments and revised the manuscript accordingly. A detailed point-by-point response to each reviewer and editor comment has been uploaded as a separate author response file.

Attachment Submitted filename: PLOS One reviewer.docx

# Peer review: Decision Letter 2

**DOI:** 10.1371/journal.pone.0353704.r005

**Authors:** Bijit Biswas

29 Jun 2026

Trends and determinants of smokeless tobacco use among Indian women: Evidence from three rounds of the National Family Health Survey 2005-2021

PONE-D-25-25520R2

Dear Dr. Sonu Goel,

We’re pleased to inform you that your manuscript has been judged scientifically suitable for publication and will be formally accepted for publication once it meets all outstanding technical requirements.

Within one week, you’ll receive an e-mail detailing the required amendments. When these have been addressed, you’ll receive a formal acceptance letter and your manuscript will be scheduled for publication.

An invoice will be generated when your article is formally accepted. Please note, if your institution has a publishing partnership with PLOS and your article meets the relevant criteria, all or part of your publication costs will be covered. Please make sure your user information is up-to-date by logging into Editorial Manager at Editorial Manager® and clicking the ‘Update My Information' link at the top of the page. For questions related to billing, please contact billing support.

If your institution or institutions have a press office, please notify them about your upcoming paper to help maximize its impact. If they’ll be preparing press materials, please inform our press team as soon as possible -- no later than 48 hours after receiving the formal acceptance. Your manuscript will remain under strict press embargo until 2 pm Eastern Time on the date of publication. For more information, please contact onepress@plos.org.

Kind regards,

Bijit Biswas, MBBS, MD, DNB

Academic Editor

PLOS One

Additional Editor Comments (optional):

Thank you for your careful revision. The reviewers agree that the concerns raised during the previous review rounds have been satisfactorily addressed, and the manuscript is now technically sound with appropriate analyses supporting the conclusions. The study provides valuable evidence on trends and determinants of smokeless tobacco use among Indian women using nationally representative data. Prior to publication, please ensure full compliance with PLOS ONE's data sharing policy and verify that the Data Availability Statement meets journal requirements.

Reviewers' comments:

Reviewer's Responses to Questions

**Comments to the Author**

1. If the authors have adequately addressed your comments raised in a previous round of review and you feel that this manuscript is now acceptable for publication, you may indicate that here to bypass the “Comments to the Author” section, enter your conflict of interest statement in the “Confidential to Editor” section, and submit your "Accept" recommendation.

Reviewer #3: All comments have been addressed

**********

2. Is the manuscript technically sound, and do the data support the conclusions?

The manuscript must describe a technically sound piece of scientific research with data that supports the conclusions. Experiments must have been conducted rigorously, with appropriate controls, replication, and sample sizes. The conclusions must be drawn appropriately based on the data presented.

Reviewer #3: Yes

**********

3. Has the statistical analysis been performed appropriately and rigorously?

Reviewer #3: Yes

**********

4. Have the authors made all data underlying the findings in their manuscript fully available?

The PLOS Data policy requires authors to make all data underlying the findings described in their manuscript fully available without restriction, with rare exception (please refer to the Data Availability Statement in the manuscript PDF file). The data should be provided as part of the manuscript or its supporting information, or deposited to a public repository. For example, in addition to summary statistics, the data points behind means, medians and variance measures should be available. If there are restrictions on publicly sharing data—e.g. participant privacy or use of data from a third party—those must be specified.

Reviewer #3: Yes

**********

5. Is the manuscript presented in an intelligible fashion and written in standard English?

PLOS ONE does not copyedit accepted manuscripts, so the language in submitted articles must be clear, correct, and unambiguous. Any typographical or grammatical errors should be corrected at revision, so please note any specific errors here.

Reviewer #3: Yes

**********

6. Review Comments to the Author

Please use the space provided to explain your answers to the questions above. You may also include additional comments for the author, including concerns about dual publication, research ethics, or publication ethics. (Please upload your review as an attachment if it exceeds 20,000 characters)

Reviewer #3: (No Response)

**********

7. PLOS authors have the option to publish the peer review history of their article (what does this mean?). If published, this will include your full peer review and any attached files.

If you choose “no”, your identity will remain anonymous but your review may still be made public.

**Do you want your identity to be public for this peer review?**  For information about this choice, including consent withdrawal, please see our Privacy Policy.

Reviewer #3: **Yes:**  Abdul Azim Abdul Razak

**********

# Editor report: Acceptance letter

**DOI:** 10.1371/journal.pone.0353704.r006

**Authors:** Bijit Biswas

PONE-D-25-25520R2

PLOS One

Dear Dr. Goel,

I'm pleased to inform you that your manuscript has been deemed suitable for publication in PLOS One. Congratulations! Your manuscript is now being handed over to our production team.

At this stage, our production department will prepare your paper for publication. This includes ensuring the following:

* All references, tables, and figures are properly cited

* All relevant supporting information is included in the manuscript submission,

* There are no issues that prevent the paper from being properly typeset

You will receive further instructions from the production team, including instructions on how to review your proof when it is ready. Please keep in mind that we are working through a large volume of accepted articles, so please give us a few days to review your paper and let you know the next and final steps.

Lastly, if your institution or institutions have a press office, please let them know about your upcoming paper now to help maximize its impact. If they'll be preparing press materials, please inform our press team within the next 48 hours. Your manuscript will remain under strict press embargo until 2 pm Eastern Time on the date of publication. For more information, please contact onepress@plos.org.

You will receive an invoice from PLOS for your publication fee after your manuscript has reached the completed accept phase. If you receive an email requesting payment before acceptance or for any other service, this may be a phishing scheme. Learn how to identify phishing emails and protect your accounts at https://explore.plos.org/phishing.

If we can help with anything else, please email us at customercare@plos.org.

Thank you for submitting your work to PLOS ONE and supporting open access.

Kind regards,

PLOS ONE Editorial Office Staff

on behalf of

Dr. Bijit Biswas

Academic Editor

PLOS One
